# ‘Obesity’ is healthy for cetaceans? Evidence from pervasive positive selection in genes related to triacylglycerol metabolism

**DOI:** 10.1038/srep14187

**Published:** 2015-09-18

**Authors:** Zhengfei Wang, Zhuo Chen, Shixia Xu, Wenhua Ren, Kaiya Zhou, Guang Yang

**Affiliations:** 1 Jiangsu Key Laboratory for Biodiversity and Biotechnology, College of Life Sciences, Nanjing Normal University, Nanjing 210023, China

## Abstract

Cetaceans are a group of secondarily adapted marine mammals with an enigmatic history of transition from terrestrial to fully aquatic habitat and subsequent adaptive radiation in waters around the world. Numerous physiological and morphological cetacean characteristics have been acquired in response to this drastic habitat transition; for example, the thickened blubber is one of the most striking changes that increases their buoyancy, supports locomotion, and provides thermal insulation. However, the genetic basis underlying the blubber thickening in cetaceans remains poorly explored. Here, 88 candidate genes associated with triacylglycerol metabolism were investigated in representative cetaceans and other mammals to test whether the thickened blubber matched adaptive evolution of triacylglycerol metabolism-related genes. Positive selection was detected in 41 of the 88 candidate genes, and functional characterization of these genes indicated that these are involved mainly in triacylglycerol synthesis and lipolysis processes. In addition, some essential regulatory genes underwent significant positive selection in cetacean-specific lineages, whereas no selection signal was detected in the counterpart terrestrial mammals. The extensive occurrence of positive selection in triacylglycerol metabolism-related genes is suggestive of their essential role in secondary adaptation to an aquatic life, and further implying that ‘obesity’ might be an indicator of good health for cetaceans.

Obesity is currently considered as a major health challenge, and hundreds of thousands of individuals are dying from obesity-related chronic diseases^1^,^2^. Obesity is primarily attributed to the excess storage of triacylglycerol (TAG) once TAG synthesis (esterification) exceeds its breakdown (lipolysis)^3^. TAG in adipose tissue serves as the major energy storage form in mammals^4^, and a proper capacity for TAG storage in adipocytes is important for normal metabolic regulation. Therefore, the assessment of diversifying mechanisms and selection pressures influencing the evolution of genes closely related to TAG metabolism can potentially provide valuable information for the treatment of obesity and other related diseases.

TAG is an ester derived from glycerol and three fatty acids (FAs)^5^, and several genes and signaling pathways are involved in its synthesis, lipolysis, and metabolic regulation. FA arises from adipocyte using two major routes: *de novo* lipogenesis from non-lipid precursors or uptake of FA from the plasma^6^. FAs are transported through the plasma membrane by several FA transporters: fatty acid translocase (FAT/CD36), fatty acid transport protein (FATP) and fatty acid binding protein, and plasma membrane (FABPpm/GOT2)^6^. Glycerol and FAs are then transformed into glycerol-3P and fatty acyl-CoA, which are used for TAG synthesis. The synthesis of phosphatidic acid and its subsequent conversion to TAG are catalyzed by several enzymes, e.g., glycerol-3-phosphate acyltransferase (GPAT) and diacylglycerolacyl transferase (DGAT)^4^. During periods of energy demand, TAG is rapidly mobilized by the hydrolytic action of lipases [desnutrin/adipose triglyceride lipase (ATGL); hormone-sensitive lipase (HSL); and monoglyceride lipase (MGLL)] that releases free fatty acids, which in turn are utilized by other organs to meet the body’s energy requirements^7^,^8^. A series of genes [e.g., Liver X-activated receptor (LXR), perilipin (PLIN), and phosphodiesterase 3B (PDE3B)] have been demonstrated to play an important role in regulating lipogenic and lipolytic processes^4^,^9^. In addition, some genes [e.g., cell death-inducing DNA fragmentation factor-α-like effector A (CIDEA) and apolipoprotein B (APOB)] have also been associated with TAG metabolism^10^,^11^. Therefore, these genes are widely recognized as candidates for controlling TAG storage conditions by changing the expression pattern and protein sequence, and a number of genes closely related to lipid metabolism have been identified to be under adaptive evolution at the genome level^12^,^13^.

Cetaceans (whales, dolphins, and porpoises) are a highly specialized group of mammals that evolutionarily transformed from a fully terrestrial quadruped to an obligate aquatic from approximately 53–56 million years ago (Ma)^14^. During this evolutionary transition, energy reserves and the maintenance of body temperature were the most critical challenges that these species encountered^15^,^16^. For example, the thickness of the blubber (the specialized hypodermis) in whales is approximately 20 cm, which is 10-fold greater than that of other artiodactyls species^17^. The blubber of cetaceans, which comprises TAG as its most important component, is dynamic and multifunctional, and acts as a metabolic energy storage site^18^, contributes to positive buoyancy^19^,^20^, provides thermal insulation^21^, supports locomotion, and increases swimming efficiency by streamlining the body surface^22^,^23^. However, the evolutionary mechanisms and driving force for the formation and maintenance of the thickened blubber in cetaceans have not been well explored to date.

In the present study, the coding regions of 88 genes that represent nearly all of the signal pathway members involved in TAG metabolism in the various cetacean lineages, were investigated and compared to orthologous sequences from terrestrial mammals using both gene- and protein-level approaches. The goal of the present study was to test whether evolutionary changes in these TAG metabolism-related genes were associated with their transition from land to water, and to determine the molecular mechanism underlying the cetacean blubber thickening during this adaptive process.

## Results

Of the 88 TAG metabolism-related genes examined in the present study, 41 key members of these genes were amplified in 5 cetacean species (Supplementary Table S1). Unfortunately, 10 genes (i.e., ACSL1, AGPAT5, AGPAT6, FABP1, FABP2, FABP6, PPARA, PPARD, PPARG, and SREBF2) were successfully amplified only in the finless porpoise (*Neophocaena phocaenoides*) and Omura’s baleen whale (*Balaenoptera omurai*), and ACSL4 was successfully amplified only in the finless porpoise (*Neophocaena phocaenoides*), despite various experimental optimizations.

### Positive selection of TAG metabolism-related genes in cetaceans

#### All-mammals dataset

**Figure 1:**
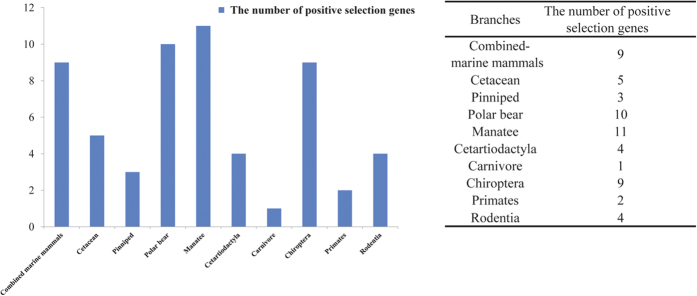
Comparison of the number of genes involved in TAG metabolism showing evidence of positive selection in mammals .

To investigate the impact of positive Darwinian selection in the 88 TAG metabolism-related genes, we used likelihood models of coding sequence evolution^24^ implemented in Codeml of the PAML package^25^. The branch-site model was used to test for positive selection in individual codons for the lineage leading to the common ancestor of each marine mammal groups (cetaceans: branch *a*; pinniped: branch *v*; polar bear: branch *w*; manatee: branch *aa* in Supplementary Fig. S1), the branch of combined marine mammals and the lineages of other groups (i.e., cetartiodactyls, carnivora, chiroptera, primates, and rodentia) across the mammalian phylogeny (Supplementary Fig. S1). Interestingly, evidence for positive selection was detected in 9 (APOB, ACSS1, AGPAT5, DGAT1, HSL1, MLXIPL, PLCB2, PLCE1, and PLIN3) of the 88 TAG metabolism-related genes examined of the combined marine mammal branches, 5 (APOB, DGAT1, MGLL, MOGAT1, and SERTAD2) genes in cetaceans, 3 (APOB, FAS, and GNPAT) genes in pinniped, 10 (APOB, DGKB, DGKZ, DGKI, FATP4, HSL, PLCB2, PLCB3, PLCH2, and PPARD) genes in polar bear and 11 (APOB, ACSL1, FAS, FATP2, MOGAT1, MOGAT2, LPL, PLCB2, PLCE1, PLIN4, and PNLIP) genes in manatee (Fig. 1 & Supplementary Table S2), which suggested the convergent evolution of TAG metabolism-related genes for the marine mammals during their adaptation to the aquatic environment. However, we also found some genes in the terrestrial groups to be positively selected, i.e. 4 genes in cetartiodactyla, 1 gene in carnivora, 9 genes in chiroptera, 2 genes in primates, and 4 genes in rodentia (Fig. 1 & Supplementary Table S2).

To further test if similar patterns of evolution occurred to the marine mammal groups, we reconstructed ancestral nodes and mapped amino acid changes along four marine mammal branches within 28 positively selected genes totally identified. Sixty-eight statistically significant (*P* < 0.05) parallel/convergent nonsynonymous amino acid substitutions were identified in the 14 of these 28 genes across two of the marine mammal lineages (Supplementary Table S3). In addition, 10, 21, 7, 2, 13, and 17 parallel/convergent mutations were found between branch pairs *a* vs *v*, *a* vs *aa*, *w* vs *a*, *w* vs *aa*, *w* vs *v*, and *v* vs *aa* in 4, 8, 3, 1, 2, and 8 genes, respectively. More importantly, the lineages leading to the common ancestor of pinniped, polar bear and manatee shared two amino acid changes (L316H & E331Q) in ACSL1.

#### Cetaceans-only dataset

**Figure 2:**
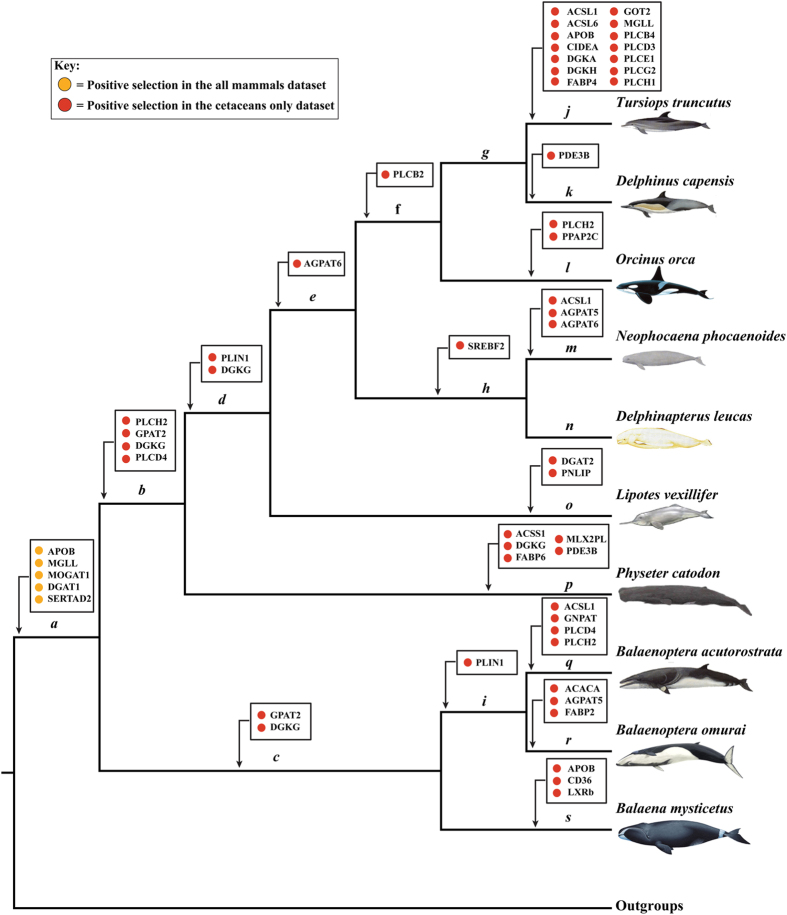
Positive selected genes related to TAG metabolism in cetaceans are mapped to a proposed phylogenetic tree. Colored circles indicated the type of positive selection identified in the present study. Branches a–s in the tree are used in the branch-site models tests. Pictures of the cetacean representative members on the right of the tree are drawn by Professor Kaiya Zhou.

The cetaceans-only dataset consisting of 88 TAG metabolism-related genes was further used to determine the selection pattern in the interior nodes of cetaceans. Of these, 35 genes were determined to be under significant positive selection along branches *b*-*s* in Fig. 2 using the branch-site models (Supplementary Table S4). A pair of site models (M8a *vs*. M8)^26^,^27^ were also used to test whether specific codons in the TAG metabolism-related genes underwent positive selection, and 23 genes were determined to have undergone positive selection in the cetaceans-only dataset, where the LRTs of the site model were statistically significant (Table 1 and Supplementary Table S5). In combination with results from above branch-site and site models, it showed that 43 genes were totally detected to be under positive selection in cetaceans by PAML.

Furthermore, the fixed-effects likelihood (FEL) and random-effects likelihood (REL) models were employed to confirm the selection pattern of TAG metabolism-related genes in cetaceans, and 26 genes were determined to be positively selected in cetaceans by the Datamonkey web server (Table 2 and Supplementary Table S6). The protein-level approach implemented in TreeSAAP^28^ identified a series of putative positively selected sites from 39 genes in cetaceans (Table 2 and Supplementary Table S6), 24 (ACSL1, ACSL5, ACSL6, ACSS1, AGPAT6, CD36, DGAT1, DGKA, DGKQ, FABP4, FATP2, GNPAT, GOT2, GPAT2, MOGAT1, MOGAT2, PDE3B, PLCB2, PLCD4, PLCH2, PLCZ1, PLIN1, PNLIP, and PPAP2C) of which were also detected by both PAML (branch-site models & site models) and Datamonkey web server (REL & FEL) methods. In addition, AGPAT5 and PLCE1 were determined to have undergone positive selection by both PAML and Datamonkey web server, whereas 15 genes (ACACA, APOB, CIDEA, DGAT2, DGKG, DGKH, FABP2, FATP3, LXRb, MGLL, PLCB4, PLCD3, PLCG2, PLCH1, and SREBF2) were detected using PAML and TreeSAAP (Table 2 and Supplementary Table S6).

**Figure 3:**
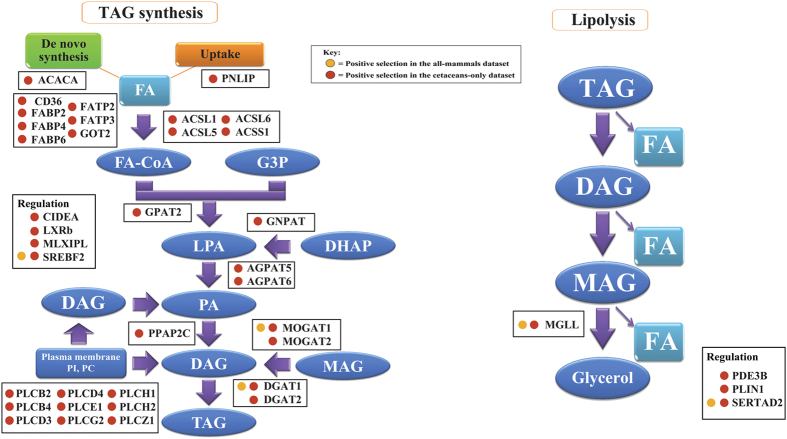
Mapping of the positively selected genes on the signal pathway of triacylglycerol synthesis and hydrolysis. Colored circles indicate the type of positive selection identified in the present study. FA, fatty acid; CoA: Coenzyme A; G3P, glycerol-3-phosphate; LPA, lysophosphatidic acid; DHAP, dihydroxyacetone-P; PA, phosphatidicacid; DAG, diacylglycerol; PI, phosphatidylinositol; PC, phosphatidylcholine; MAG, monoacylglycerol; TAG, triacylglycerol.

To summarize the above results from the analyses of two datasets using different methods, 44 genes were identified to have undergone positive selection in cetaceans (Figs 2 and 3; Tables 1 and 2), and 5 (APOB, DGAT1, MGLL, MOGAT1, and SERTAD2) of these were subjected to strong positive selection in the common ancestor of the cetacean, whereas no selection was observed in terrestrial mammalian lineages such as cetartiodactyls, carnivores, and primates. It is generally accepted that a positively selected site is more reliable if it can be supported by two or more different methods. Of the positively selected genes, 41 were validated by at least two methods, thus they were used in the subsequent analyses. In terms of the functions of these 41 genes, 8 genes (ACACA, CD36, FABP2, FABP4, FATP2, FATP3, GOT2, and PNLIP) were involved in FA synthesis or transport, 26 genes (ACSL1, ACSL5, ACSL6, ACSS1, AGPAT5, AGPAT6, DGAT1, DGAT2, DGKA, DGKG, DGKH, DGKQ, GNPAT, GPAT2, MOGAT1, MOGAT2, PLCB2, PLCB4, PLCD3, PLCD4, PLCE1, PLCG2, PLCH1, PLCH2, PLCZ1, and PPAP2C) in TAG biosynthesis, and 1 gene (MGLL) in TAG lipolysis, and 6 genes (APOB, CIDEA, LXRb, PDE3B, PLIN1, and SREBF2) were associated with the regulation of TAG metabolism.

#### Spatial distribution of the positively selected sites in the protein structures

Functional domains of each TAG metabolism-related gene were further examined to determine the functional significance of the putative positively selected sites, and the results showed that most identified positively selected sites were localized in or close to the functional regions in the protein structures of the 24 genes (Supplementary Fig. S2 & Supplementary Table S7). In 5 of these genes (i.e., ACSL1, CD36, DGAT2, GPAT2, and MOGAT1), 20 positively selected sites were located within the protein transmembrane domain of the corresponding genes. In addition, most of the positively selected sites were localized in the topological domain of ACSL1, ACSL5, ACSL6, CD36, DGAT1, DGAT2, FATP2, GPAT2, and SREBF2. For CD36, DGKA, DGKG, DGKH, DGKQ, LXRb, PLCD3, PLCD4, PLCE1, PLCH2, and PNLIP, some positively selected amino acids were located in the disulfide bond, glycosylation, zinc finger domain, substrate binding domain, ligand-binding domain, and so on, respectively (Supplementary Table S7). Furthermore, one positively selected site (29) was located in the nuclear localization signal motif of the FABP4 gene, and positively selected sites 440, 651, and 727 in the PDE3B gene were located in the catalytic regions, respectively (Supplementary Table S7).

## Discussion

Cetaceans have a thick layer of blubber with a mean thickness of 98.4 ± 18.4 mm^29^. The thick blubber layer surrounding the cetacean body can comprise more than 30% of their body mass, and it is far greater than the 4–8% dissectible adipose found in the general healthy wild animal^30^. However, the genetic basis underlying the blubber thickening remains poorly explored. The present study therefore presents the first systematic investigation of TAG metabolism-related genes of representative cetaceans and closely related terrestrial mammals. Wide and strong signals of positive selection were detected in genes related to TAG synthesis or regulation of TAG synthesis, which could in turn provide novel insights into the evolution of blubber thickening in cetaceans.

The positive selection in cetacean DGAT1 and DGAT2 genes detected in the present study supports the morphological evidence that whales have thicker blubbers than those of other artiodactyl species^17^. The DGAT-catalyzed synthesis of TAG is the final and rate-limiting step in TAG formation (Fig. 3), and it is believed that DGAT is a key factor in controlling the production of triglycerides and fatty acids, as well as plays a key modulatory role in animal fat deposition^31^. Therefore, the observed positive selection in the DGAT1 and DGAT2 genes in cetaceans is suggestive of an enhanced capability for TAG formation. In addition, evidence of positive selection has been shown for other genes involved in the remaining pathways of TAG synthesis (Fig. 3). Effective uptake of free fatty acids can accelerate the expansion of adipocyte dimensions when lipids accumulate^32^. Acetyl-coA carboxylase 1 (ACC1), which play important roles in the *de novo* synthesis of FAs^6^,^33^, underwent positive selection in cetaceans (Table 2 and Fig. 3). Furthermore, PNLIP, the primary pancreatic TAG lipase^34^, was also subjected to positive selection across cetaceans, suggesting the essential role of FAs from the hydrolysis and absorption of long-chain triglyceride FAs from food during TAG synthesis in cetaceans. CD36, FABP2 (adipocytes lipid binding protein), FABP4, FATP2, FATP3, and GOT2, which are used in facilitating and regulating the transport of FAs across the plasma membrane, were determined to be under significant positive selection in cetaceans, which suggests that cetaceans might have acquired an enhanced capacity for FAs transport to maintain fat deposition. In additional, a series of essential enzymes involved in different steps of TAG biosynthesis pathways, i.e., ACSL1, ACSL5, ACSL6, ACSS1, AGPAT5, AGPAT6, DGKA, DGKG, DGKH, DGKQ, GNPAT, GPAT2, MOGAT1, MOGAT2, PLCB2, PLCB4, PLCD3, PLCD4, PLCE1, PLCG2, PLCH1, PLCH2, PLCZ1, and PPAP2C^18^, were determined to have undergone positive selection in cetaceans (Table 2 and Fig. 3), which in turn suggests that cetaceans have possess an effective ability to enhance their TAG synthesis during their adaptation to a fully aquatic life. Remarkably, some regulatory genes related to TAG synthesis were also determined to be under positive selection (Fig. 3), which may then imply a complex molecular mechanism of cetacean blubber thickening. Liver X-activated receptor b (LXRb), an important protein that controls the amount of cellular SREBP-1c, underwent significant positive selection in cetaceans (Table 2 and Fig. 3). The expression of FAS and mtGPAT can be greatly increased once SREBP-1c is overexpressed, which in turn results in an increase in FA synthesis and TAG deposition^10^,^11^. Furthermore, CIDEA, which enhances lipid droplet size when ectopically expressed in preadipocytes and in turn favors cellular lipid accumulation^35^, also presented evidence of positive selection in cetaceans. Positive selection of these genes might therefore play an important role in promoting cetacean blubber thickening.

Positive selection of different enzymes involved in cetacean TAG synthesis might directly explain the molecular basis of cetacean blubber thickening. Interestingly, besides from the TAG synthesis-related genes, MGLL, an important major lipases involved in lipolysis^36^, was also under strong positive selection, as indicated by the results of nucleotide- and protein- level analyses (Table 2 and Fig. 3). These findings therefore suggest that lipolysis was advanced to a certain degree in cetaceans, and the metabolic rate of cetaceans might have been increased to compensate for the energy shortage.

Cetaceans exhibit physiological and anatomical adaptations that allow them to rely on the lipids stored in their blubber as a source of energy during annual fasting periods^37^. During times of energy shortage, TAGs stored in lipid droplets are hydrolyzed to FAs and glycerol via lipolysis for subsequent use by other organs, and dolphins had more rapid release of non-esterified FAs than other mammals^38^. In addition, the water derived from metabolism, particularly lipolysis, is considered to be the primary sources of fresh water for cetaceans^39^. MGLL hydrolyzes MAG, thereby producing glycerol and FAs^6^. Therefore, positive selection of MGLL might be helpful for rapid release FAs to producing energy and to more efficiently extracting water in cetaceans, which in turn allows them to survive annual fasting periods with limited amounts of TAG stored in body tissues.

Cetacean blubber was vertically stratified and each blubber layer performs a different function, with the stable outer layer used for structural support and the more variable inner layer used for energy storage^40^. A suitable blubber thickness is essential for cetaceans to adapt to the aquatic environment. However, when in an emaciated state during the fasting period, the dual roles of the blubber in providing insulation and storing metabolic energy are in direct conflict^20^. The absence of a mechanism to regulate or control the utilization of stored TAGs in the blubber as an energy resource can result in the excessive use of TAGs during the fasting period, which in turn might greatly decrease the thickness of blubber and further weaken the activities of thermoregulation, buoyancy control, streamlining, and locomotion that are essential for aquatic life. Amazingly, evidence of positive selection in two genes involved in the regulation of lipolysis (i.e., PDE3B and PLIN1) was detected in cetacean-specific lineages, which maybe a mechanism for avoiding excessive lipolysis. PDE3B is a very powerful regulator of adipocyte lipolysis that is triggered by a decrease in cAMP levels^41^. The importance of PDE3B in suppressing adipocyte lipolysis has been demonstrated in PDE3B null mice^42^. PLIN1 is a key member of the PLIN family that encode for proteins that cover the lipid droplets in adipocytes, as well as regulate the coordination of lipid storage and utilization in various cell types^36^. It has been suggested that PLINs play an important role in inhibiting lipolysis when it is unphosphorylated^43^. Considering the function of these genes in regulating and inhibiting TAG lipolysis, positive selection of these genes suggests that cetaceans have evolved an enhanced capacity for inhibiting unrestricted lipolysis and finely control the fatty acid content of blubber, and therefore is important for the maintenance of a suitable thickness of the blubber layer.

Blubber’s thickness and lipid content extensively varies across cetaceans. For example, the lipid content of harbor porpoise blubber ranges from 76% to 88%, whereas that of minke whales ranges from 42% to 96%^20^. The thermal conductance capacity of the blubber is highly dependent on both its conductive quality and quantity (i.e., thickness), and the difference in the thickness and lipid content of the blubber among cetaceans might be the result of their adaptation to different aquatic habitats. Therefore, positively selected genes are distributed throughout almost the entire cetacean phylogeny, from the most common ancestral branch of cetaceans to the terminal branches (Fig. 2). A series of positively selected sites observed in 24 genes were localized in or near the functional regions on the crystal structure of the corresponding genes (Supplementary Fig. S2 and Supplementary Table S7), which indicates the ongoing adaptive evolution of cetacean TAG metabolism-related genes. It is also reasonable to assume that different cetaceans require a fine blubber layer, which has driven the relevant genes to evolve in response to continuous changes in the aquatic environment since their origin and subsequent diversification in waters across the globe.

Remarkably, APOB, the primary lipid-binding protein of chylomicrons and low-density lipoproteins (LDL)^44^, was determined to have undergone positive selection in the lineage leading to the common ancestor of each marine mammalian groups (i.e., cetaceans, pinnipeds, polar bear, manatee) and the branch of the combined marine mammals (Supplementary Table S2), which is consistent with the previous findings that strong adaptive selection was detected in the APOB gene of polar bears^45^. In cetaceans, blood-based LDL levels are much lower than humans^46^, and APOB may play an important role in the LDL redistribution in the blubber of cetaceans. In addition, blubber is a critical component of mammalian adaptation to the aquatic environment, and cetaceans and other aquatic animals show remarkable similarities in blubber structure and function^37^. Comparative genomic analyses have found that convergent amino acid substitutions were widespread throughout the genome, and PNLIP and PLIN4 genes were identified to be positively selected respective along the combined marine mammal branch and the cetacean branch in Foote *et al*. (2015)^47^. In addition, 68 parallel/convergent nonsynonymous amino acid substitutions were identified in 14 positively selected genes detected. These results further supported the adaptive evolution of cetacean TAG metabolism-related genes, and suggest that cetaceans and other marine mammals have apparently been under similar pressure for adipose tissue development and fatty acid metabolism during their adaptation to the aquatic environments. However, further investigation on the functional verification of TAG metabolism-related genes in cetaceans and other marine mammals is necessary in the future to determine their role in aquatic adaptations. Moreover, some positively selected genes were also detected in the terrestrial groups, suggesting the importance of TAG metabolism during their adaptation to various environments, and further research should be focused on this interesting phenomenon to interpret the roles of TAG metabolism in terrestrial mammals.

The present study is the first comprehensive and systematic analysis of the molecular genetic basis of blubber thickening in cetaceans. Wide and strong positive selection was detected in several genes involved in TAG synthesis, lipolysis, and regulation, which is concordant with the important functions of cetacean blubber in thermoregulation, buoyancy control, streamlining, metabolic energy storage, and locomotion. Interestingly, some regulation genes that inhibit lipolysis also showed significant evidence of positive selection, which suggests that cetaceans have evolved an enhanced capacity for inhibiting unrestricted lipolysis, particularly during fasting. This study provides novel insights into the effective and complex mechanism of maintaining a suitable blubber layer thickness in cetaceans, and also implies that ‘obesity’ might be an indicator of good health for cetaceans, compared to that determined in humans, which has been strongly associated to various chronic diseases.

## Methods

### Samples and DNA sequencing

Five cetacean species (two mysticetes and three odontocetes): common minke whale, Omura’s whale, Beluga, Finless porpoise, and long-beaked common dolphin were sequenced during this study. All 5 cetacean samples used in the present study were collected from dead individuals in the wild, and sampling was conducted systematically in accordance with all ethical guidelines and legal requirements in China. The protocol of this study was approved by the Institutional Review Board of Nanjing Normal University (NNU). Voucher specimens were preserved at Jiangsu Key Laboratory for Biodiversity and Biotechnology, College of Life Sciences, Nanjing Normal University (NNU), China.

Total genomic DNA was extracted from muscle with a standard phenol/chloroform procedure followed by ethanol precipitation^48^. The DNA integrity was checked by 1% agarose gel electrophoresis. Primers were designed for the conserved regions based on an alignment of genomic data from the cow (*Bos taurus*) (http://asia.ensembl.org/Bos_taurus/Info/Index) and bottlenose dolphin (*Tursiops truncatus*) (http://asia.ensembl.org/Tursiops_truncatus/Info/Index). All PCR amplification were conducted using a BioRAD PTC-200 with 2 × EasyTaq PCR SuperMix (TransGen Biotech) and the following profile: 34 cycles at 94 °C for 5min, 94 °C for 30 s, 53 °C−59 °C for 30 s, and 72 °C for 30 s, followed by a 10 min extension at 72 °C. The amplified PCR products were purified and sequenced in both directions using an ABI 3730 automated genetic analyzer. Three to five repeated amplification for each gene were conducted and resequenced to confirm its sequence. The specificity of these newly generated sequences was examined by comparison with the published nucleotide database at GenBank by BLAST (NCBI).

### Source of data and primary treatments

Forty-one TAG metabolism-related genes (Supplementary Table S1) were sequenced in the five cetacean species mentioned above and the newly sequences were deposited in GenBank under accession numbers KR135543-KR135734. The exons of each gene were sequenced and concatenated before being analyzed together. Only high-quality and high-integrity sequences were used in the analysis. In addition, gene sequences from other mammals [including bottlenose dolphin (*Tursiops truncatus*)] were searched and primarily downloaded from the OrthoMaM^49^, and gene sequences derived from the genomes of baiji (*Lipotes vexillifer*), common minke whale (*Balaenoptera acutorostrata scammoni*), killer whale (*Orcinus orca*), sperm whale (*Physeter macrocephalus*), seal (*Leptonychotes weddellii*), walrus (*Odobenus rosmarus divergens*), manatee (*Trichechus manatus latirostris*), polar bear (*Ursus maritimus*) (ftp://ftp.ncbi.nlm.nih.gov/genomes/) and bowhead whale (*Balaena mysticetus*) (http://www.bowhead-whale.org/) were also used to test for positive Darwinian selection. A total of 88 TAG metabolism-related genes (Supplementary Table S1) and 23–50 species from representatives mammalian lineages (i.e., Cetaceans, Artiodactyla, Chiroptera, Rodentia, Carnivora, and Primates) were analyzed in the present study (Supplementary Table S8 and Supplementary Table S9). Nucleotide sequences of each gene examined and their deduced amino acid sequences were aligned separately using MUSCLE 3.8^50^ and MEGA 5.0^51^, and manually adjusted with GeneDoc.

### Molecular evolutionary analyses

The codon-based maximum likelihood models implemented in CODEML program in PAML 4.7^25^ were applied to estimate the rates of synonymous (*dS*) and nonsynonymous substitutions (*dN*), as well as *dN/dS* ratio (omega, ω). The non-synonymous to synonymous rate ratio ω indicates changes in selective pressures, where ω = 1, ω < 1, and ω > 1 correspond to neutral evolution, purifying, and positive selection, respectively. The well-supported phylogeny of Laurasiatheria^52^ and Primates^53^ was used as the input tree in all analyses (Tree file: Supplementary Fig. S1).

Positive selection was detected using branch-site model A, in which ω can vary among sites along specific lineages^54^. Modified branch-site model A (test 2) was performed for every gene in each foreground lineage, which facilitated the analysis of datasets, including all mammals (branches *a*, *t*-*aa* in Supplementary Fig. S1) or cetaceans only (branches b-s in Supplementary Fig. S1). To identify the probabilities of sites under positive selection in each gene for the cetacean species examined, site models were implemented where ω could vary among sites. All the positively selected sites in site models were identified by using Bayes Empirical Bayes (BEB) analysis^25^ with posterior probabilities of ≥0.80. The likelihood ratio test (LRT) statistic (2ΔL) approximates to a Chi-square distribution and was used to compare nested likelihood models. In addition, the improved statistical methods in Datamonkey web server^55^, which computed nonsynonymous and synonymous substitutions at each codon position, was used to further evaluate the selection. Sequences of each gene in cetaceans-only dataset were analyzed by using two distinct models, namely, fixed-effect likelihood (FEL) and random effect likelihood (REL).The FEL model estimates the ratio of *dN/dS* on a site-by-site basis, without assuming an *a priori* distribution across sites. The REL model first fits a distribution of rates across sites and then infers the substitution rate for individual sites. Sites with *P* values < 0.1 for FEL, and Bayes factor >50 for REL were considered as candidates under positive selection^55^.

Some studies have suggested that the ML method of evaluating positive selection might produce false-positive results even when no positively selected sites exist^56^ or when positively selected sites and negatively selected sites are mixed^57^. Further support for the PAML results were obtained using a complementary protein-level approach implemented in TreeSAAP^28^. TreeSAAP compares the magnitude of property changes of non-synonymous residues across a phylogeny and identifies specific amino acid properties that have likely been affected by positive destabilizing selection during evolutionary^58^.

### Identification of parallel/convergent sites among marine mammals

The parallel/convergent sites among marine mammals were identified according to the methods previously described^47^. In detail, we reconstructed the ancestral sequences for 26 positive selection genes using the Codeml program in PAMLv4.7. For each of the four marine mammal groups (cetaceans, pinnipeds, polar bear and manatee) the extant sequences at each position were compared to the ancestral sequence at the node corresponding to the most recent ancestor. We used the software CONVERG 2^59^ to test whether the number of observed parallel/convergent amino acid substitutions was significantly higher than that expected by chance, given the total numbers of amino acid replacements in the two evolutionary lineages under investigation. The positions of the parallel/convergent nonsynonymous amino acid substitutions that were found in positively selected genes are shown in Supplementary Table S3.

### Mapping of positively selected sites onto protein structures

To gain insights into the functional significance of the putatively selected sites, we mapped the sites under positive selection to crystal structures. The 3D structures of genes under positive selection were predicted by using the homology modeling software provided by the I-TASSER server^60^. The protein sequences of positively selected genes were derived from the common bottlenose dolphin (*Tursiops truncutus*) genome, which were obtained from the Ensembl genome database (http://www.ensembl.org/index.html). In addition, the functional information of genes identified as being under positive selection was derived from the uniprot (http://www.uniprot.org/).

## Additional Information

**Accession codes:** The 41 TAG metabolism-related genes (Supplementary Table S1) were sequenced in five additional cetacean species (two mysticetes and three odontocetes): common minke whale (*Balaenoptera acutorostrata*), Omura's whale (*Balaenoptera omurai*), Beluga (*Delphinapterus leucas*), Finless porpoise (*Neophocaena phocaenoides*), and long-beaked common dolphin (*Delphinus capensis*), and these new sequences were deposited in GenBank as accession numbers KR135543-KR135734.

**How to cite this article**: Wang, Z. *et al* .‘Obesity’ is healthy for cetaceans? Evidence from pervasive positive selection in genes related to triacylglycerol metabolism. *Sci. Rep* .**5**, 14187; doi: 10.1038/srep14187 (2015).

## Supplementary Material

Supplementary Information

Supplementary Table S2

Supplementary Table S3

Supplementary Table S4

Supplementary Table S5

Supplementary Table S6

Supplementary Table S7

Supplementary Table S8

Supplementary Table S9

## Figures and Tables

**Table 1:** M8 & M8a analysis and evidence of positive selection on 23 genes related to TAG metabolism in cetaceans.

Gene	-InL (M8a)	-InL (M8)	P value	ω value (M8)	Positively selected sites
ACSL1	3880.189837	3877.642968	0.024	1.965	100 (0.800) 169 (0.842) 183 (0.829) 254 (0.877) 310 (0.863) 333 (0.852) 374 (0.936) 657 (0.849) 661 (0.838) 662 (0.877)
ACSL5	3762.751885	3756.161056	<0.01	10.609	62 (0.855) 208 (0.883) 222 (0.983) 429 (0.845) 466 (0.994) 644 (0.838) 666 (0.975) 685 (0.858)
ACSL6	2974.997590	2972.082248	0.016	2.991	17 (0.872) 124 (0.908) 160 (0.923) 171 (0.917) 215 (0.899) 276 (0.931) 410 (0.986)
CD36	2970.142564	2958.040852	<0.01	2.388	18 (0.964) 19 (0.953) 38 (0.874) 42 (0.886) 60 (0.991) 65 (0.939) 145 (0.935) 151 (0.934) 152 (0.911) 154 (0.874) 156 (0.998) 157 (0.892) 158 (0.964) 160 (0.999) 161 (0.916) 166 (0.916) 190 (0.857) 195 (0.948) 217 (0.899) 236 (0.927) 263 (0.969) 325 (0.949) 349 (0.919) 354 (0.963) 355 (0.951) 355 (0.951) 358 (0.855) 383 (0.961) 397 (0.946) 398 (0.899) 399 (0.981) 401 (0.905) 411 (0.997) 414 (0.855) 440 (0.915) 447 (0.846) 448 (0.934) 450 (0.961) 454 (0.945) 458 (0.858) 459 (0.942) 469 (0.957) 471 (0.894) 472 (0.897) 473 (0.999)
CIDEA	1101.607445	1101.608921	0.003	1.337	
DGAT1	2107.987504	2104.943568	0.014	14.431	419 (0.964)
DGAT2	1681.978411	1684.391038	0.028	3.305	41 (0.893) 42 (0.930) 67 (0.827) 117 (0.918) 177 (0.984) 227 (0.849)
DGKA	3433.817808	3431.719676	0.041	5.593	451 (0.898) 572 (0.966) 720 (0.888)
DGKH	5595.551465	5592.410358	0.012	503.770	1202 (0.824)
DGKQ	4046.831703	4041.082855	0.001	31.380	278 (0.800) 279 (0.979) 313 (0.866) 338 (0.828)
FABP2	664.987160	663.011920	0.047	3.837	11 (0.856) 105 (0823) 26 (0.817)
FABP4	713.695341	705.734379	<0.01	7.501	24 (0.957) 29 (0.951) 33 (0.943) 41 (0.959) 53 (0.961) 57 (0.955) 86 (0.954) 122 (0.996)
FATP2	3100.854796	3098.494072	0.030	8.292	176 (0.905) 242 (0.981) 251 (0.817) 356 (0.839) 392 (0.868)
FATP3	3400.109844	3395.996365	0.004	999.000	3 (0.920) 5 (0.893) 165 (0.859) 241 (0.819) 505 (0.819) 604 (0.864) 676 (0.803)
GNPAT	3550.257763	3546.213234	0.004	2.503	23 (0.877) 176 (0.873) 178 (0.958) 205 (0.832) 214 (0.880) 434 (0.853) 441 (0.886) 462 (0.854) 466 (0.863) 477 (0.841) 583 (0.882) 664 (0.879)
GPAT2	4645.296344	4640.895446	0.003	21.420	439 (0.964) 445 (0.912) 479 (0.873) 572 (0.856) 677 (0.849)
MLXIPL	3835.065500	3832.275129	0.018	346.673	
MOGAT1	1795.790529	1789.419812	<0.01	13.111	26 (0.891) 27 (0.987) 30 (0.999) 34 (0.903) 43 (0.873) 292 (0.885)
MOGAT2	2011.219495	2001.596828	<0.01	17.931	74 (0.996) 238 (0.983) 255 (0.942) 295 (0.997)
PDE3B	4061.866626	4051.580665	<0.01	4.205	18 (0.972) 68 (0.987) 83 (0.930) 84 (0.814) 85 (0.812) 97 (0.812) 161 (0.994) 185 (0.885) 187 (0.950) 284 (0.966) 301 (0.919) 304 (0.895) 337 (0.957) 339 (0.961) 651 (0.944) 727 (0.900)
PLCZ1	2914.700506	2911.365281	0.010	49.708	340 (0.959)
PNLIP	2548.952558	2540.199574	<0.01	3.504	47 (0.890) 49 (0.859) 88 (0.974) 101 (0.824) 104 (0.849) 105 (0.927) 112 (0.911) 153 (0.869) 223 (0.835) 232 (0.972) 237 (0.970) 306 (0.994) 336 (0.838) 337 (0.859) 338 (0.893) 341 (0.995) 342 (0.859) 359 (0.888) 366 (0.868) 395 (0.887) 402 (0.846)
PPAP2C	1454.520261	1447.266740	<0.01	199.838	253 (0.823) 258 (0.847) 261 (0.855) 267 (0.863) 268 (0.931) 276 (0.924)

**Table 2:** Positive selection in 41 cetacean TAG metabolism-related genes based on the analysis of the cetacean-only dataset by PAML, DATAMONKEY, or TreeSAAP.

Gene	PAML^*^	DATAMONKEY^*^	TreeSAAP^*^
ACACA	Y	N	Y
ACSL1	Y	Y	Y
ACSL5	Y	Y	Y
ACSL6	Y	Y	Y
ACSS1	Y	Y	Y
AGPAT5	Y	Y	N
AGPAT6	Y	Y	Y
APOB	Y	N	Y
CD36	Y	Y	Y
CIDEA	Y	N	Y
DGAT1	Y	Y	Y
DGAT2	Y	N	Y
DGKA	Y	Y	Y
DGKG	Y	N	Y
DGKH	Y	N	Y
DGKQ	Y	Y	Y
FABP2	Y	N	Y
FABP4	Y	Y	Y
FATP2	Y	Y	Y
FATP3	Y	N	Y
GNPAT	Y	Y	Y
GOT2	Y	Y	Y
GPAT2	Y	Y	Y
LXRb	Y	N	Y
MGLL	Y	N	Y
MOGAT1	Y	Y	Y
MOGAT2	Y	Y	Y
PDE3B	Y	Y	Y
PLCB2	Y	Y	Y
PLCB4	Y	N	Y
PLCD3	Y	N	Y
PLCD4	Y	Y	Y
PLCE1	Y	Y	N
PLCG2	Y	N	Y
PLCH1	Y	N	Y
PLCH2	Y	Y	Y
PLCZ1	Y	Y	Y
PLIN1	Y	Y	Y
PNLIP	Y	Y	Y
PPAP2C	Y	Y	Y
SREBF2	Y	N	Y

Note: Y, positive selection was detected; N, no positive selection was detected.

^*^Results are listed in Supplementary Tables S4,5,6.
