# Supplementary material for: ‘Obesity’ is healthy for cetaceans? Evidence from pervasive positive selection in genes related to triacylglycerol metabolism
Source: Sci Rep. 2015 Sep 18;5:14187. doi: 10.1038/srep14187 (PMC4585638; doi:10.1038/srep14187)
Supplement: Supplementary Information [file srep14187-s1.pdf]

Obesity is healthy for cetaceans: Evidence from pervasive positive selection in genes related to triacylglycerol metabolism

Zhengfei Wang[1], Zhuo Chen[1, 2], Shixia Xu[1], Wenhua Ren[1],

Kaiya Zhou[1] and Guang Yang[1]*

Supplementary Table S1. The 88 genes of TAG metabolism Analyzed in this Study.

**Synthesis**		**Lipolysis**	**Regulation**
ACC1*	FABP5	AdPLA*	ABHD5
ACSL1*	FABP6*	ATGL*	ADIPOQ*
ACSL3	FABP7	HSL*	ADRP*
ACSL4*	GOT2(FABPpm)	LIPH	AMPK
ACSL5*	FAS*	LPL*	CAV1
ACSL6*	CD36(FAT)	MGLL*	CIDEA
ACSS1	FATP1*	PNLIP*	LXRa
ACSS2	FATP2*		LXRb
ACSS3	FATP3*		MLXIPL
AGPAT1*	FATP4		PDE3B*
AGPAT2	GNPAT		PLIN1*
AGPAT3*	GPAT1*		PLIN3
AGPAT4*	GPAT2*		PLIN4
AGPAT5*	MOGAT1*		PLIN5
AGPAT6*	MOGAT2*		PPARA*
DGAT1*	MOGAT3*		PPARD*
DGAT2*	PLCB1		PPARG*
DGKA	PLCB2		SERTAD2*
DGKB	PLCB3		SREBF1*
DGKE	PLCB4		SREBF2*
DGKG	PLCD3		APOB
DGKH	PLCD4		
DGKI	PLCE1		
DGKQ	PLCG1		
DGKZ	PLCG2		
FABP1*	PPAP2A		
FABP2*	PPAP2B		
FABP3	PPAP2C		
FABP4*			

* Forty-one genes were sequenced for five cetacean species (two mysticetes: common minke whale (*Balaenoptera omurai*), Omura's whale (*Balaenoptera acutorostrata*), and three odontocetes: Beluga (*Delphinapterus leucas*), Finless porpoise (*Neophocaena phocaenoides*), and Long-beaked common dolphin (*Delphinus capensis*)) in this study.

Abbreviations:

ACSL: Acyl-CoA Synthetase Long-chain family member; ACSS: Acyl-CoA Synthetase Short-chain family member; ACC1 (also known as ACACA): Acetyl-CoA Carboxylase Alpha
AGPAT: 1-Acylglycerol-3-Phosphate O-Acyltransferase; DGAT: Diacylglycerol O-Acyltransferase; DGKA: Diacylglycerol Kinase, Alpha; DGKB: Diacylglycerol Kinase, Beta; DGKG: Diacylglycerol Kinase, Gamma; DGKH: Diacylglycerol Kinase, Eta; DGKI: Diacylglycerol Kinase, Iota; DGKQ: Diacylglycerol Kinase, Theta; DGKZ: Diacylglycerol Kinase, Zeta; GNPAT: Glyceronephosphate O-Acyltransferase; GPAT: Glycerol-3-Phosphate

Acyltransferase; MOGAT: Monoacylglycerol O-Acyltransferase; FABP: Fatty Acid Binding Protein; FABPpm(also known as GOT2): Glutamic-Oxaloacetic Transaminase 2; FAS: Fatty Acid Synthase; FAT (also known as CD36): CD36 molecule (thrombospondin receptor); FATP: Solute Carrier Family 27 (Fatty Acid Transporter), member; PLCB: Phospholipase C, Beta; PLCD: Phospholipase C, Delta; PLCE: Phospholipase C, Epsilon; PLCG: Phospholipase C, Gamma; PPAP2A: Phosphatidic Acid Phosphatase type 2A; PPAP2B: Phosphatidic Acid Phosphatase type 2B; PPAP2C: Phosphatidic Acid Phosphatase type 2C; AdPLA: Adipose-Specific Phospholipase A2; ATGL: Adipose Triglyceride Lipase; HSL: Hormone-Sensitive Lipase; LIPH: Lipase, Member H; LPL: Lipoprotein Lipase; MGLL: Monoglyceride Lipase; PNLIP: Pancreatic Lipase
ABHD5: Abhydrolase Domain Containing 5; ADIPOQ: Adiponectin, C1Q and collagen domain containing; ADRP (also known as PLIN2): Adipose Differentiation-Related Protein; AMPK: AMP-activated protein kinase; CAV1: Caveolin 1, Caveolae Protein; CIDEA: Cell Death-Inducing DFFA-Like Effector A; LXRa: Liver X Receptor Alpha Protein; LXRb: Liver X Receptor Beta Protein; MLXIPL (also known as ChREBP): Carbohydrate Response Element Binding Protein; PDE3B: Phosphodiesterase 3B; PLIN: Perilipin; PPARA: Peroxisome Proliferator-Activated Receptor Alpha; PPARD: Peroxisome Proliferator-Activated Receptor Delta; PPARG: Peroxisome Proliferator-Activated Receptor Gamma; SERTAD2: SERTA domain-containing Protein 2; SREBF: Sterol Regulatory Element Binding Transcription Factor
APOB: Apolipoprotein B.

Supplementary Figure Legends

Supplementary Figure S1. A well supported phylogeny of mammals used for selective pressure analysis in PAML. Tree topologies of Laurasiatheria and primates were from Zhou et al. (2012)[52] and Perelman et al. (2011)[53], respectively. Different orders of mammals were marked with various colors. Branches a–aa in the tree are used in the branch-site models tests, and results are listed in Tables S2 and Tables S4.

Supplementary Figure S2. Spatial distribution of positively selected sites in the three-dimensional (3D) structure of 15 genes related to TAG metabolism in cetaceans.

Supplementary Figure S1

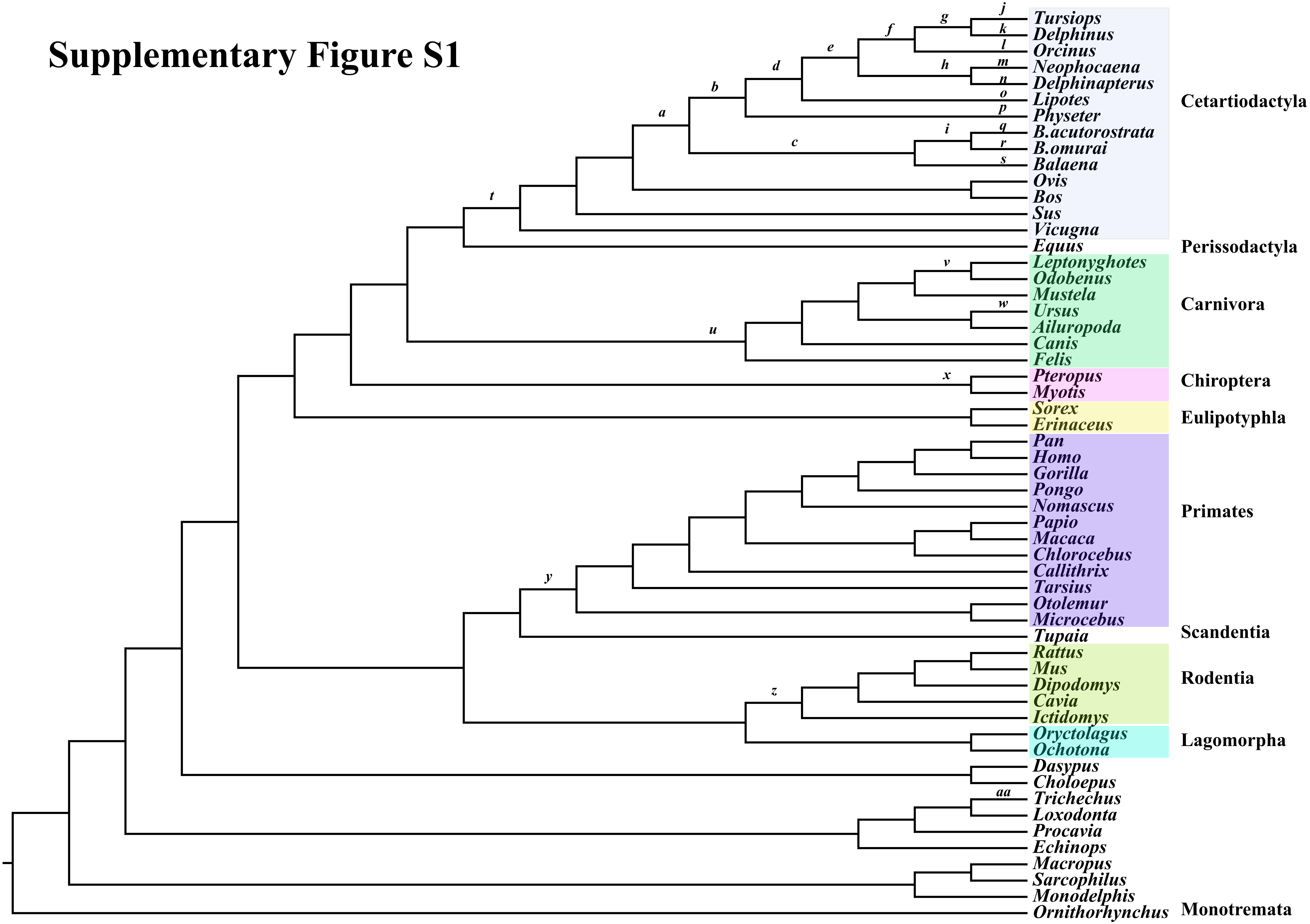

Supplementary Figure S2

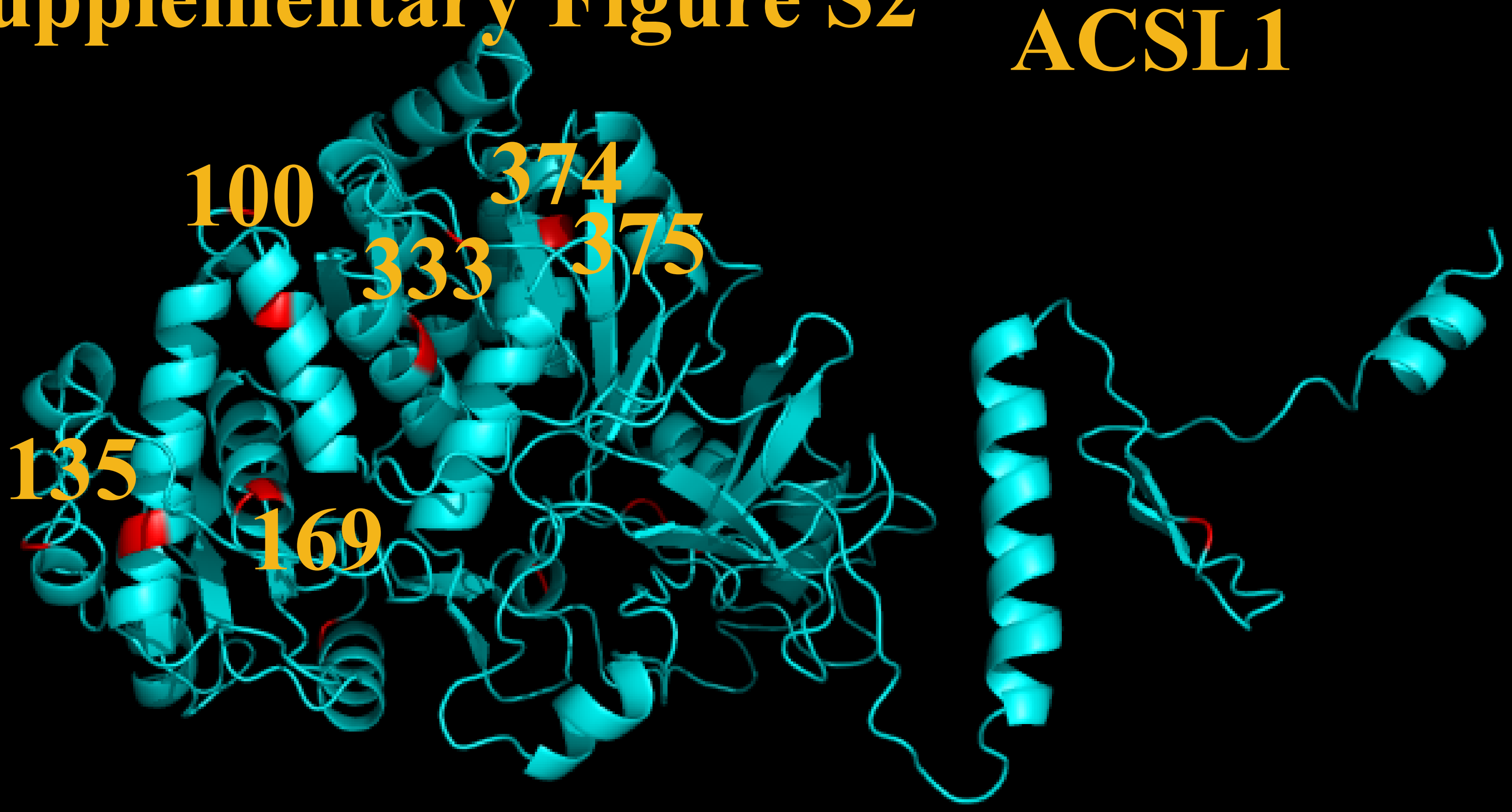

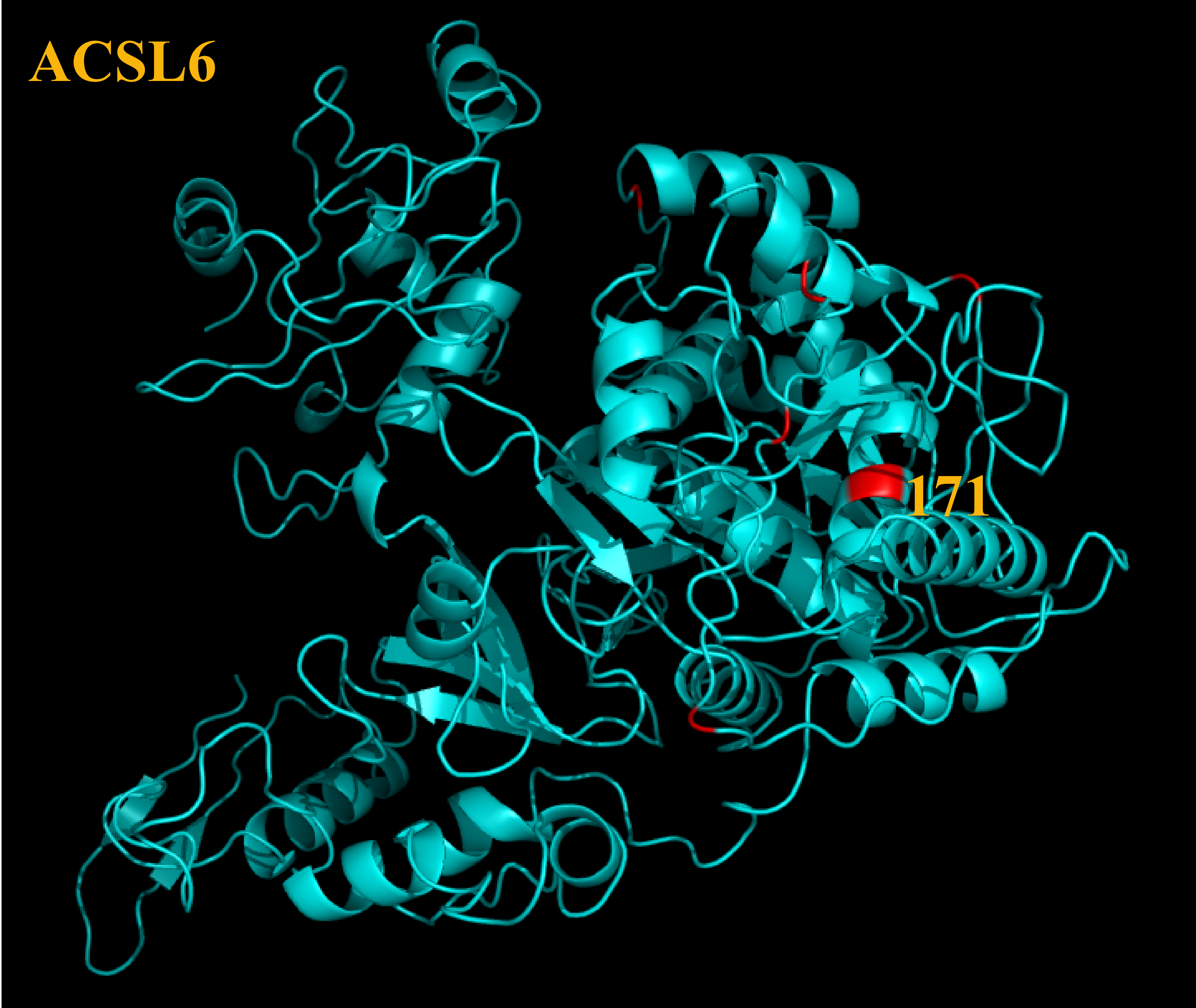
ACSL6
171

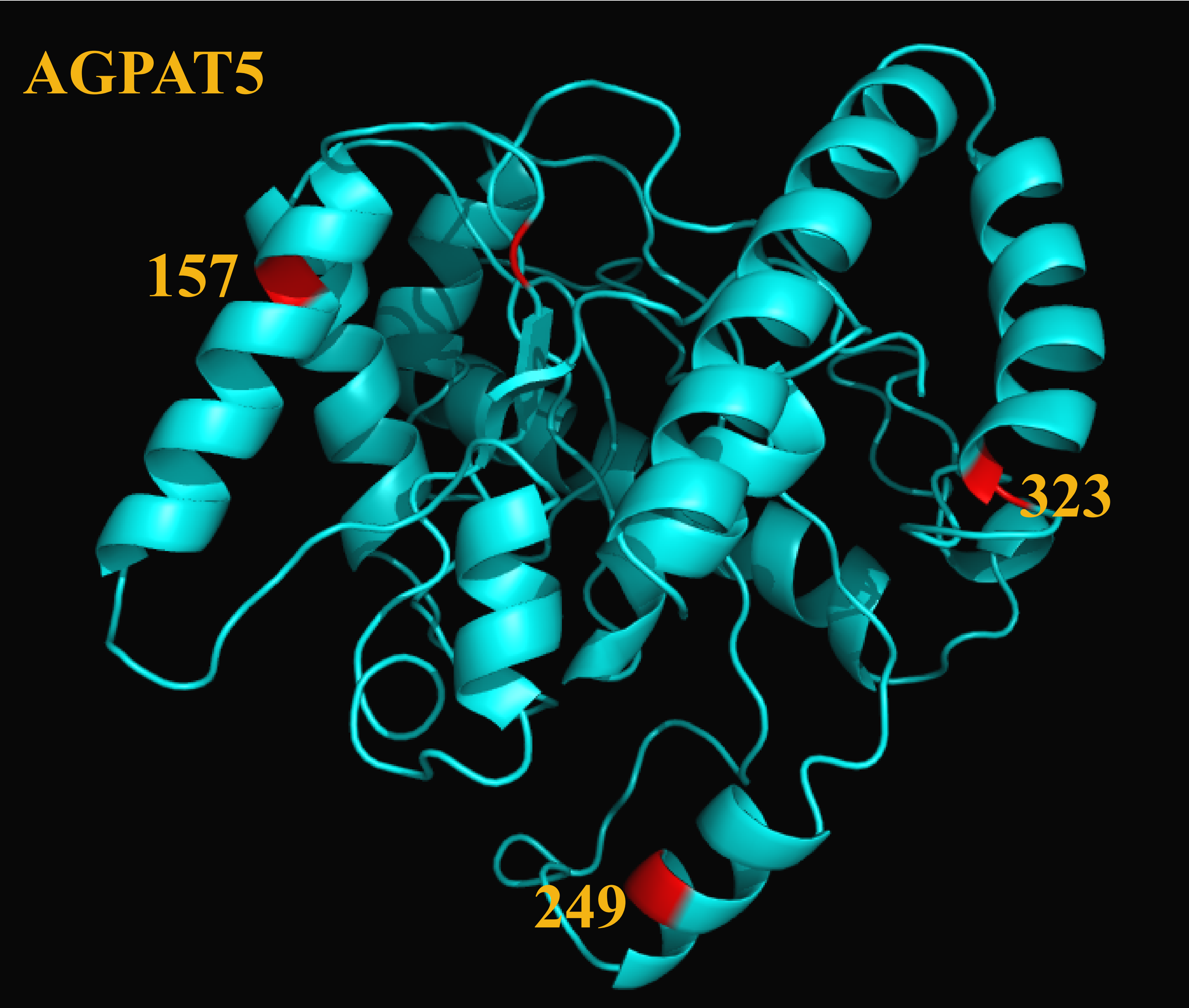
AGPAT5
157
323
249

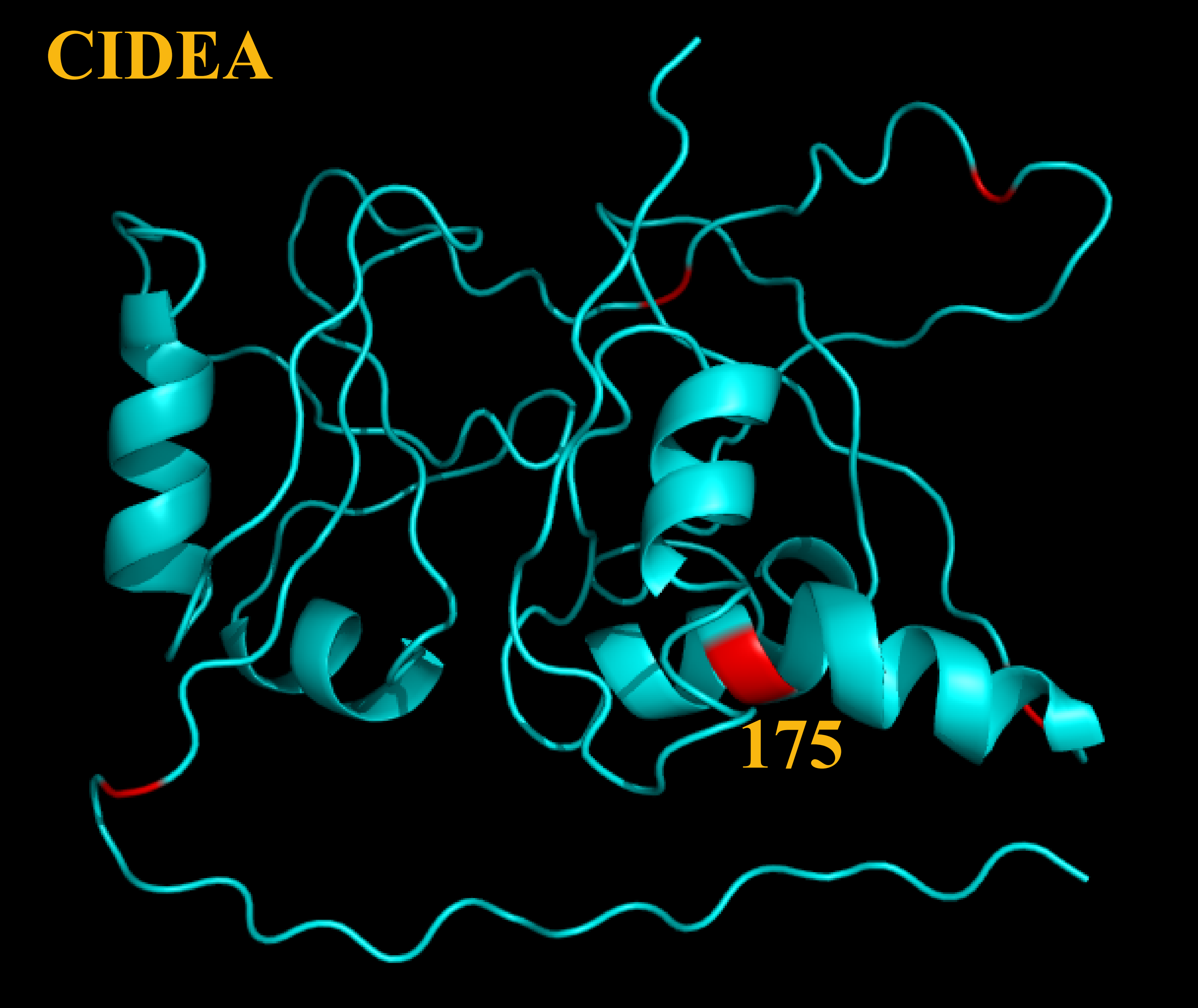
CIDEA
175

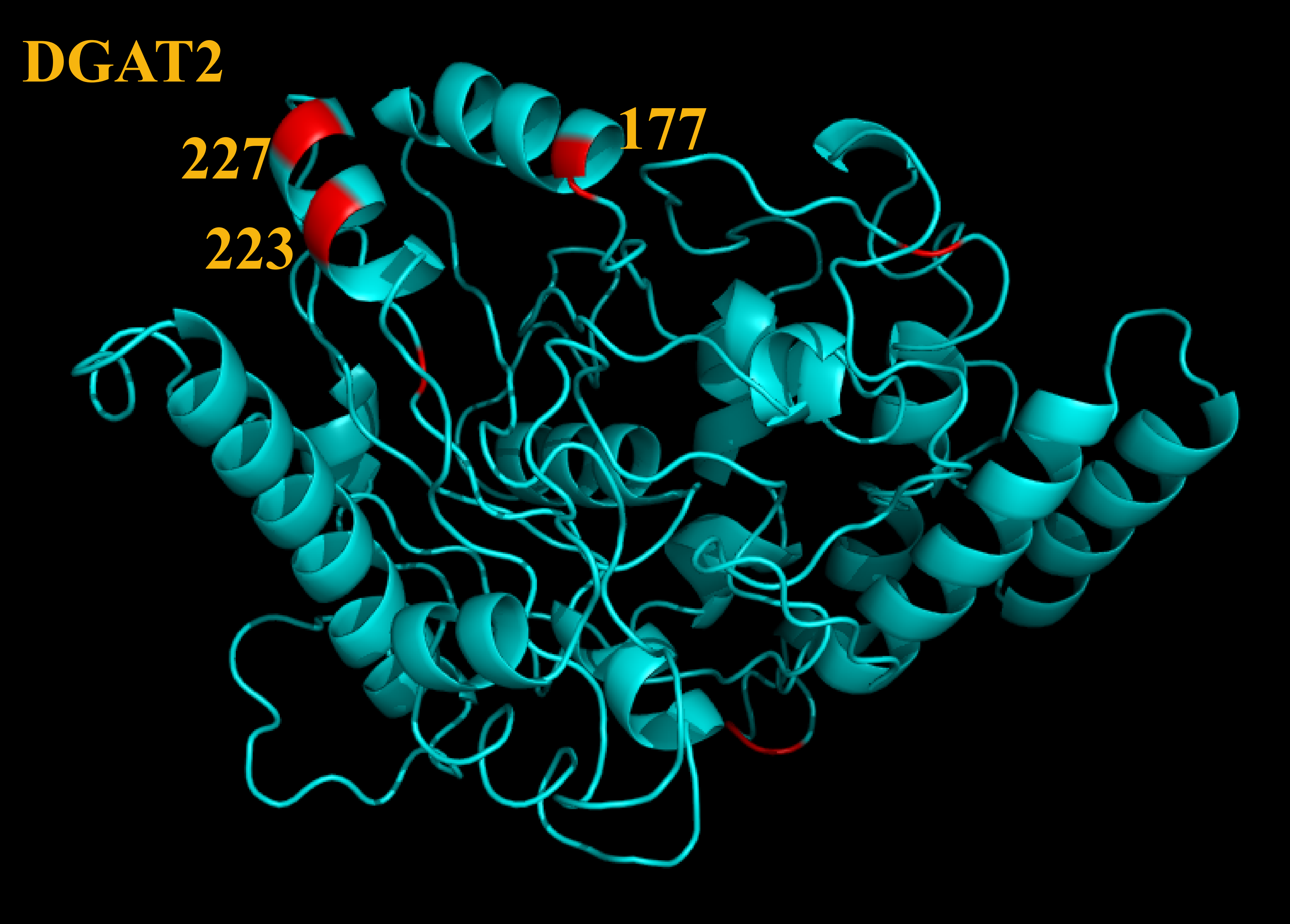
DGAT2
227
223
177

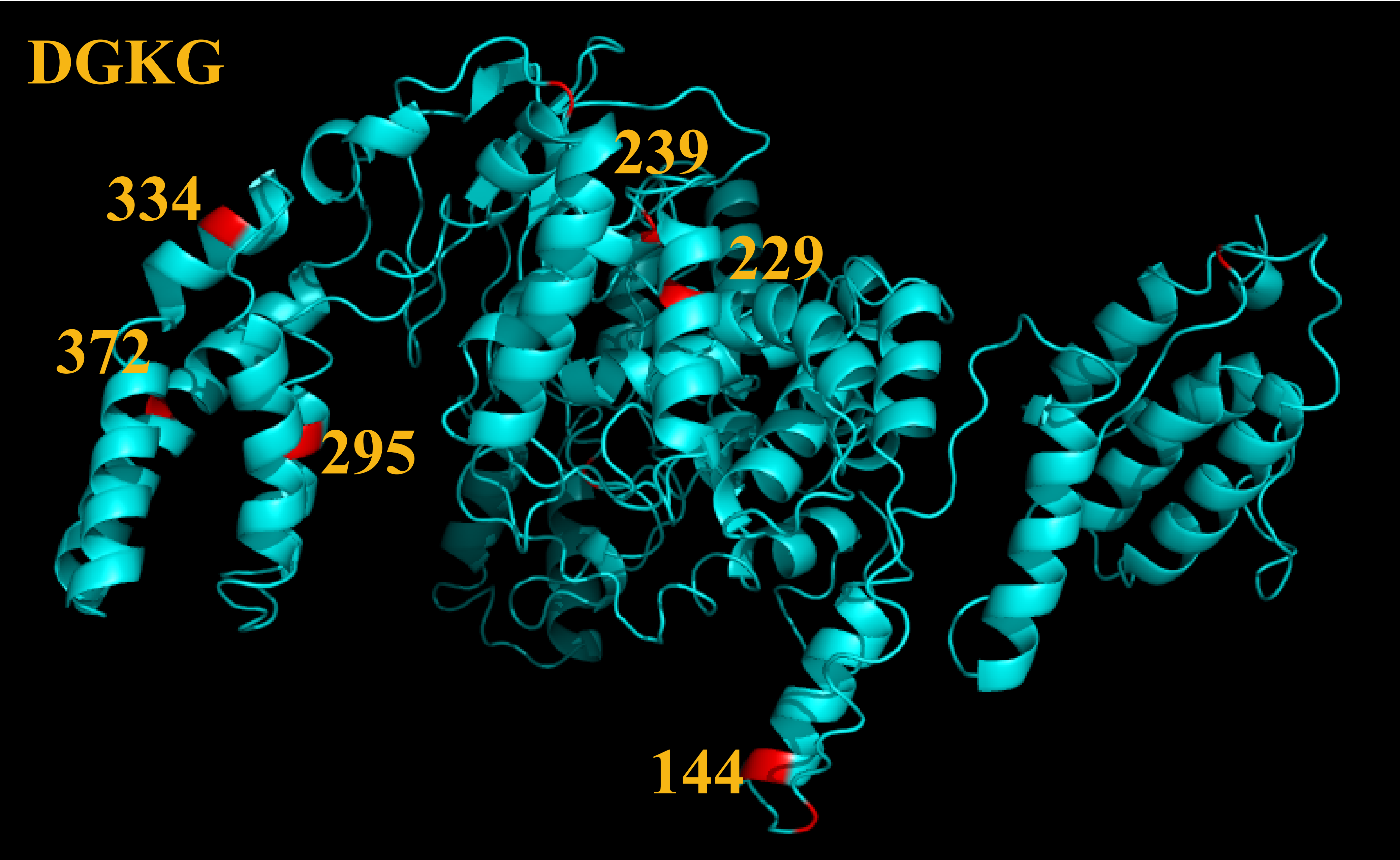
DGKG
239
334
229
372
295
144

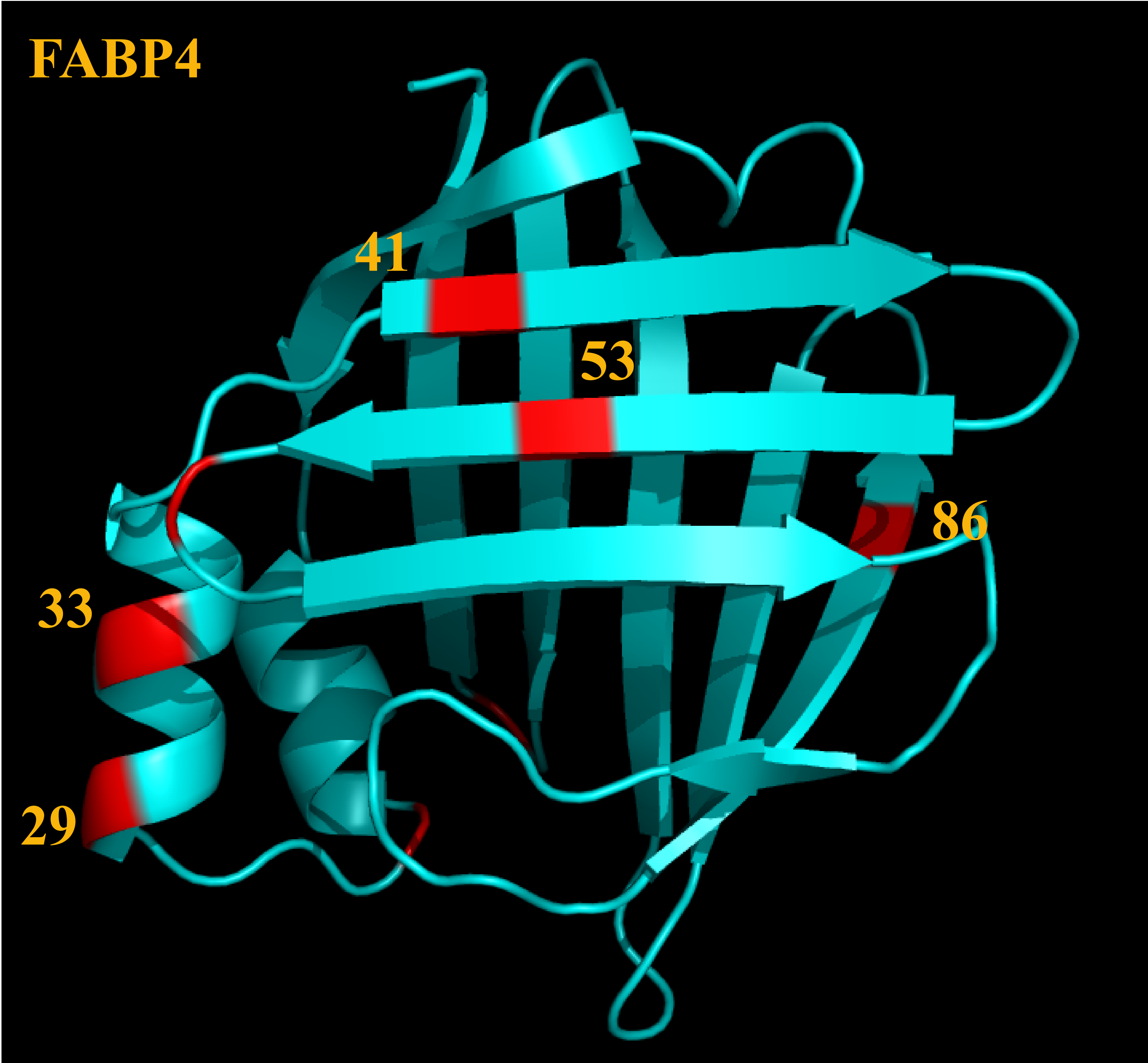
FABP4
41
53
86
33
29

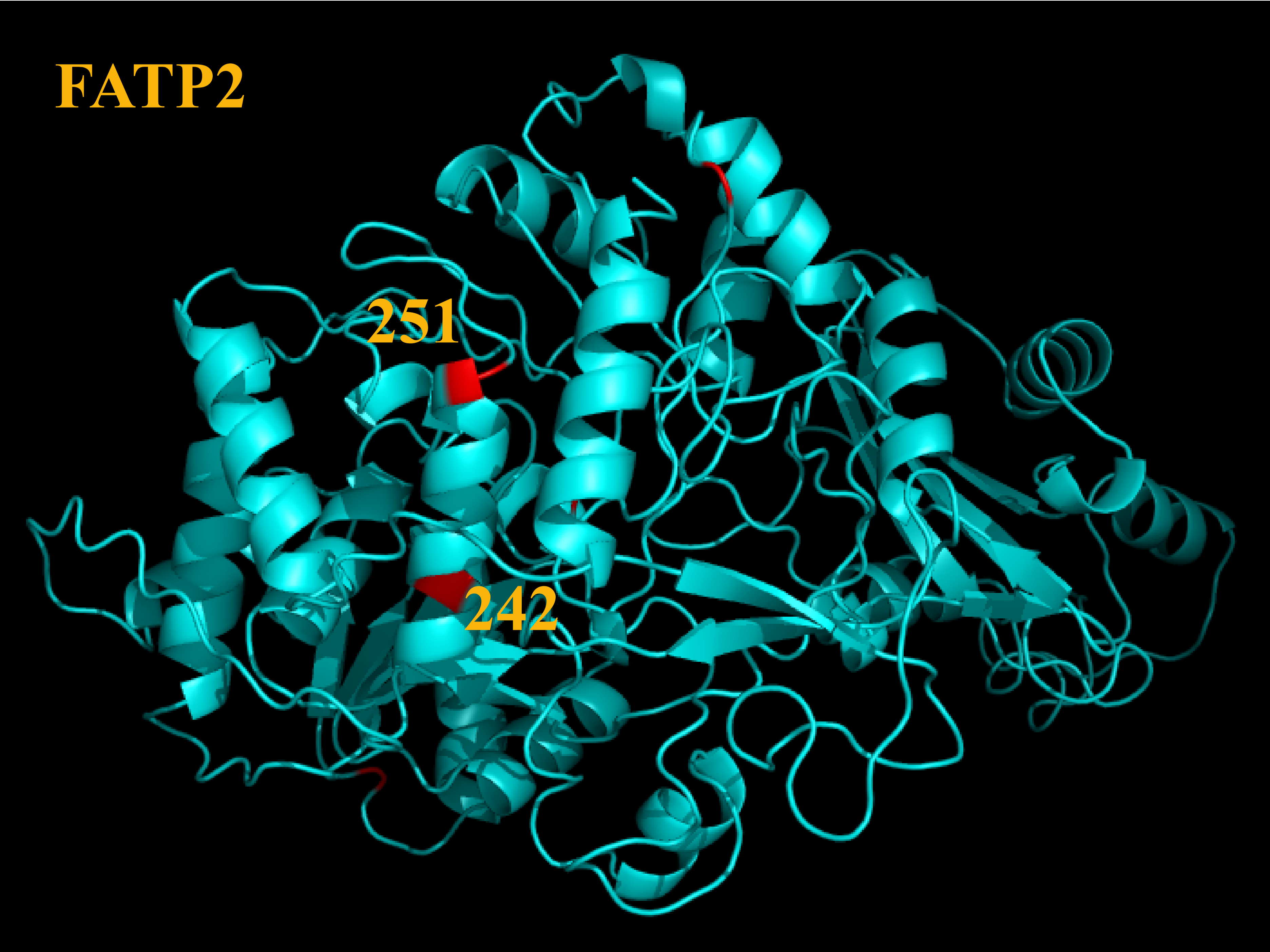
FATP2
251
242

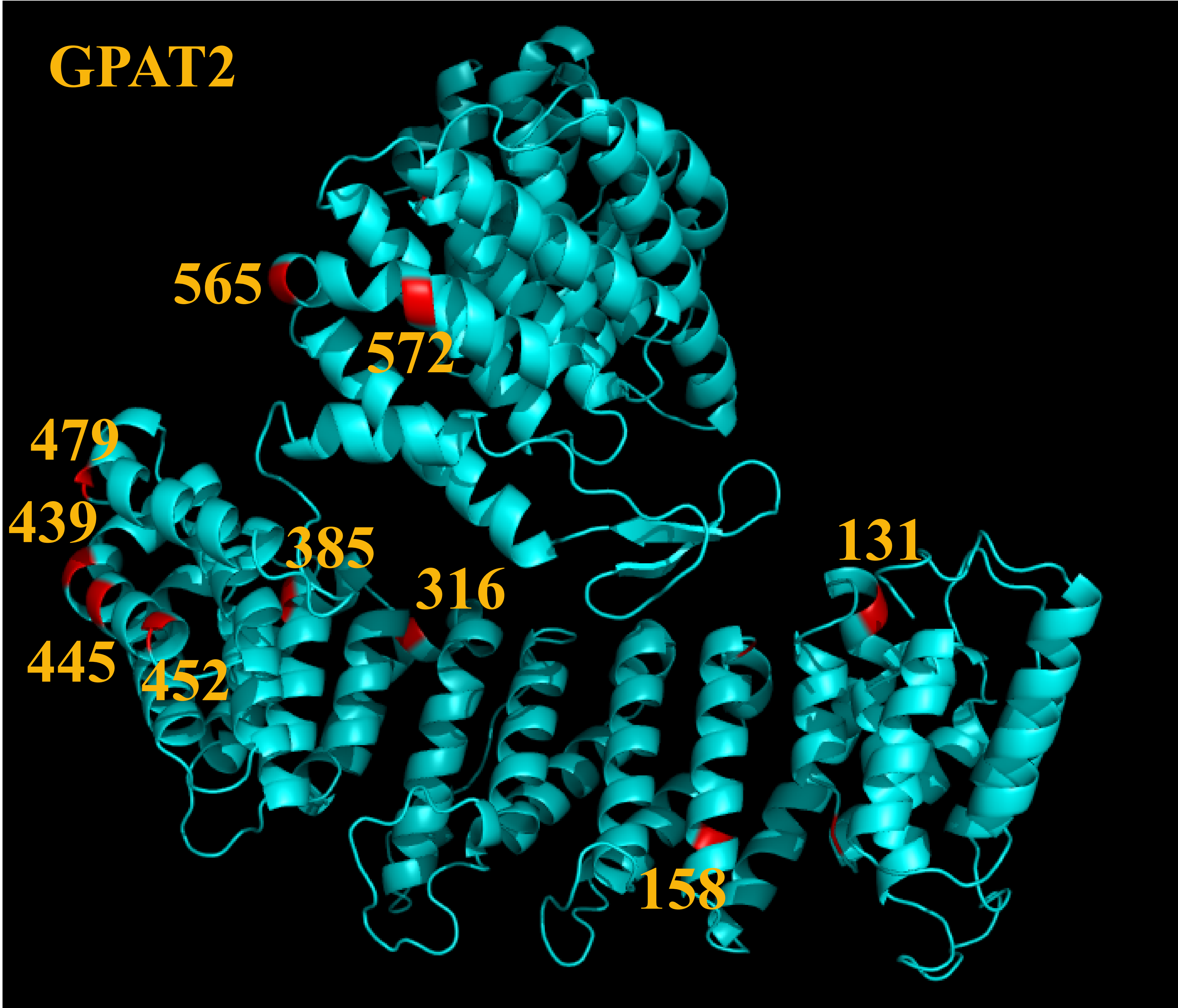
GPAT2
565
572
479
439
385
316
131
445
452
158

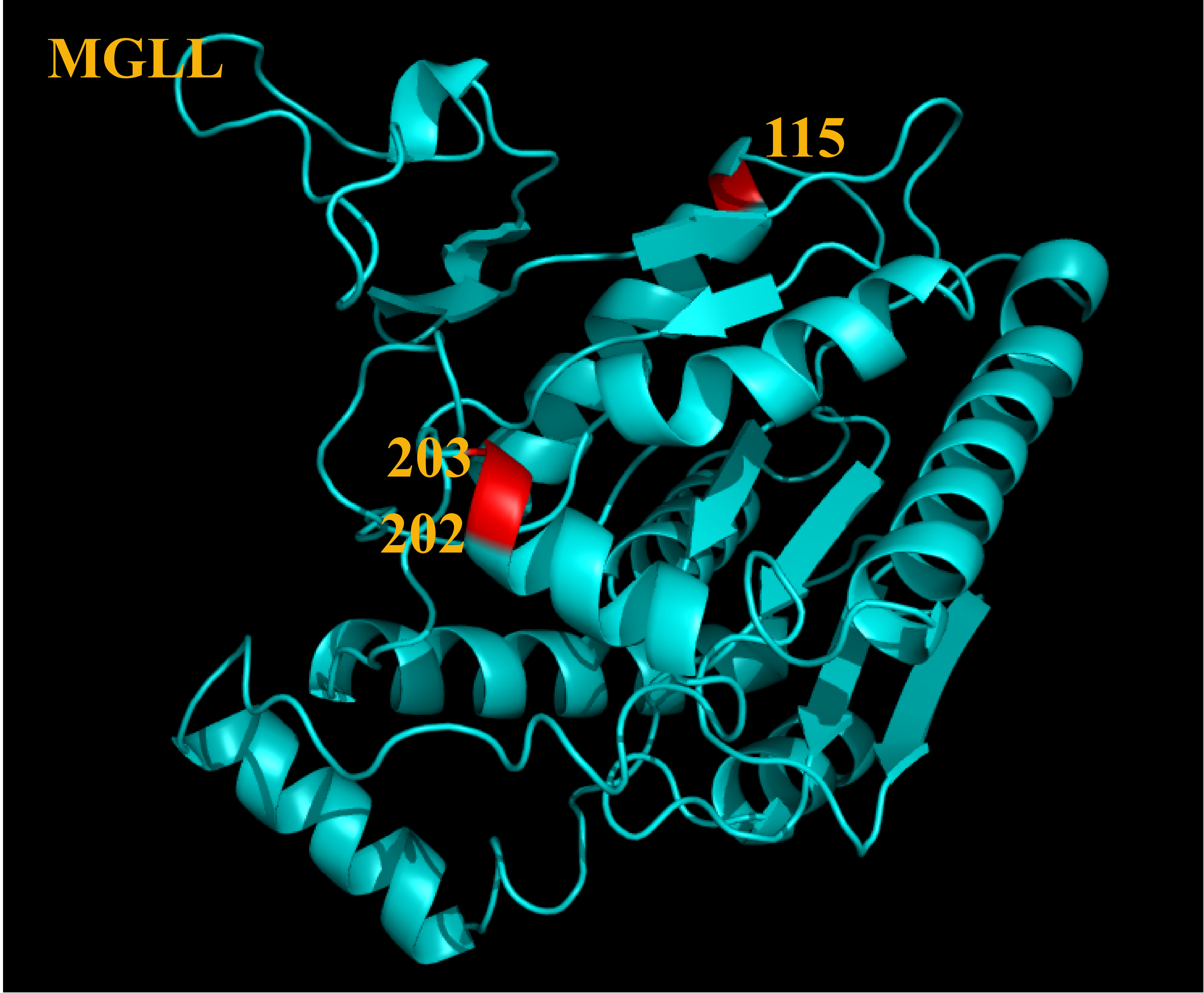
MGLL
115
203
202

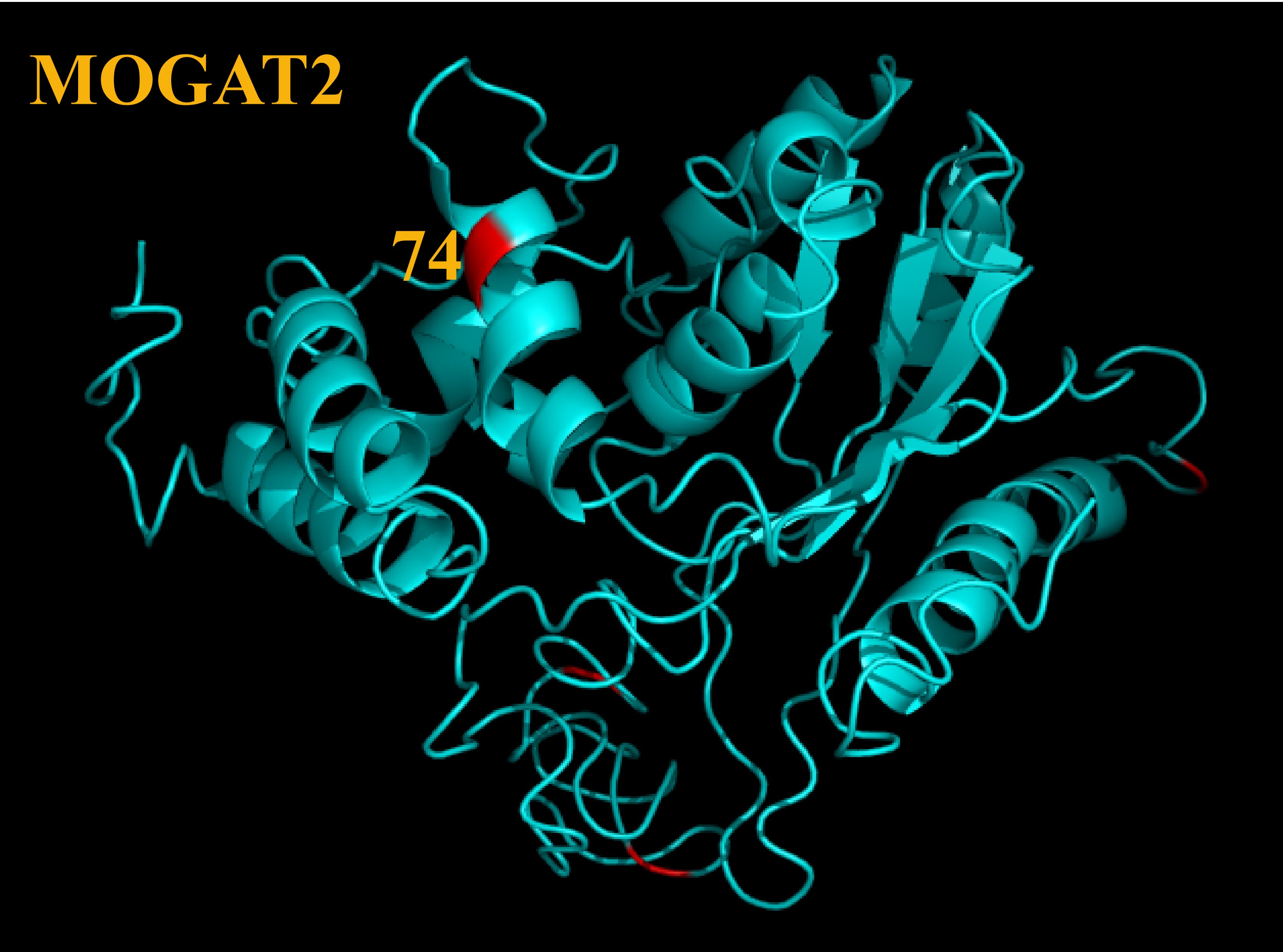
MOGAT2
74

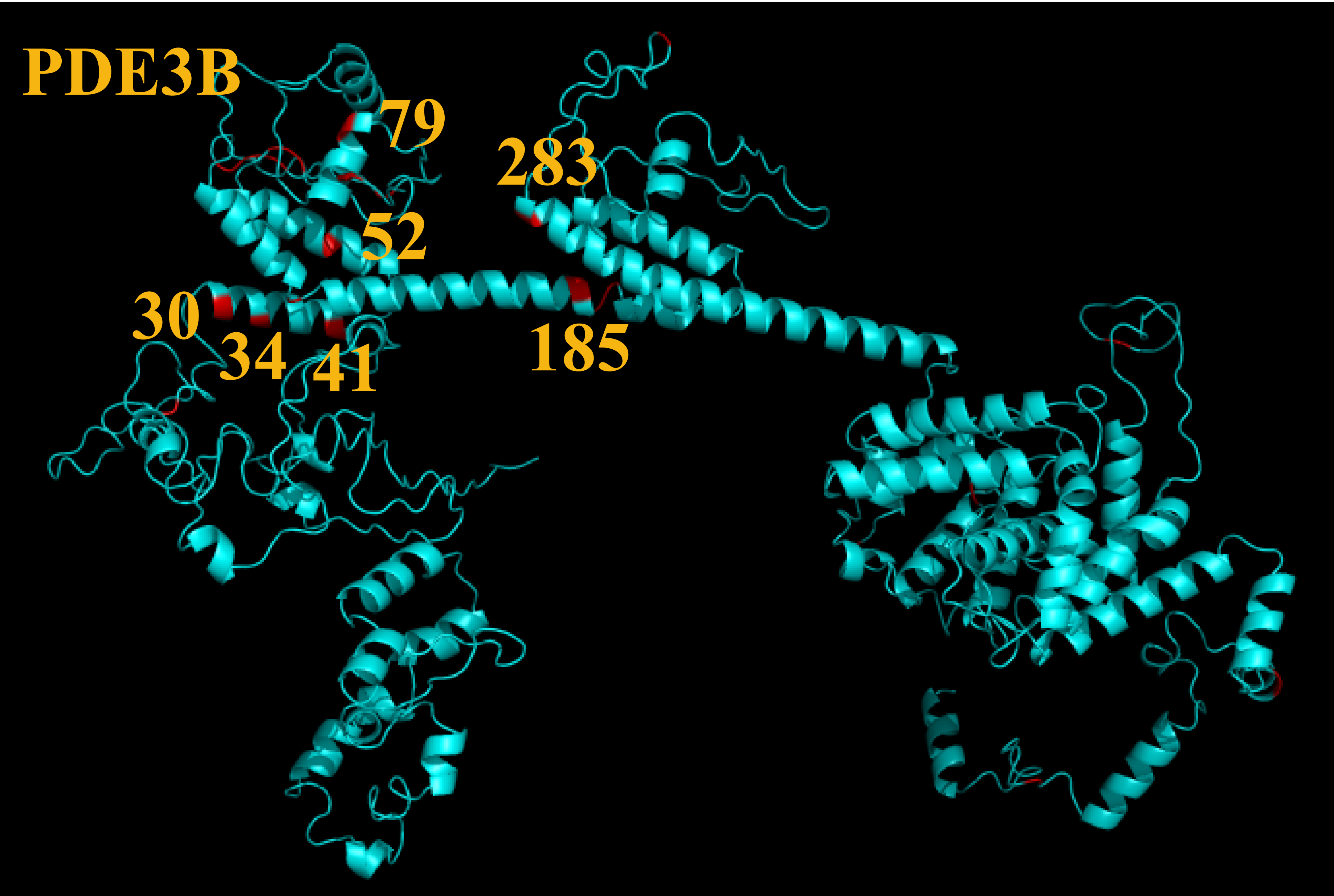
PDE3B
79
283
52
30
34
41
185

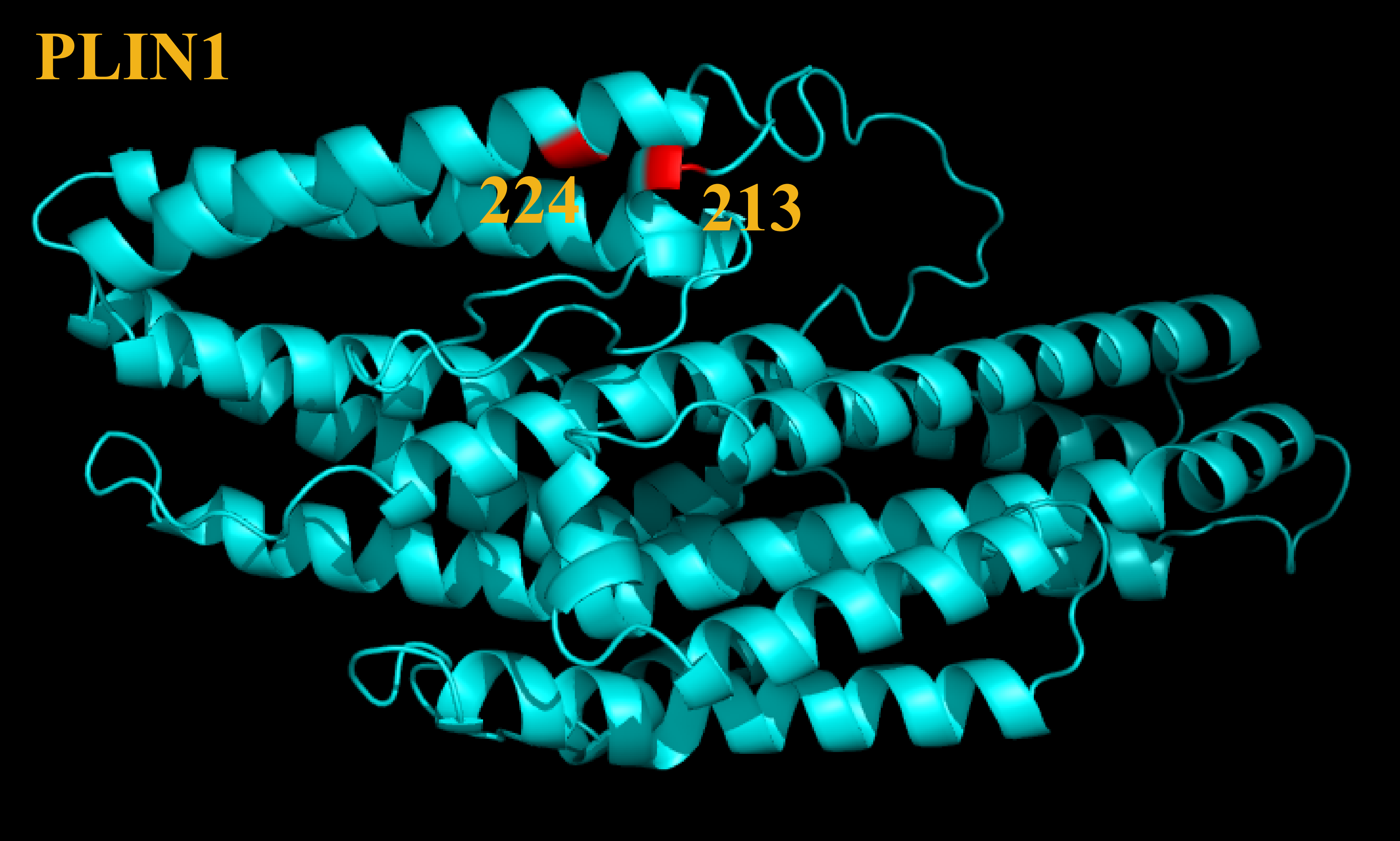
PLIN1
224
213

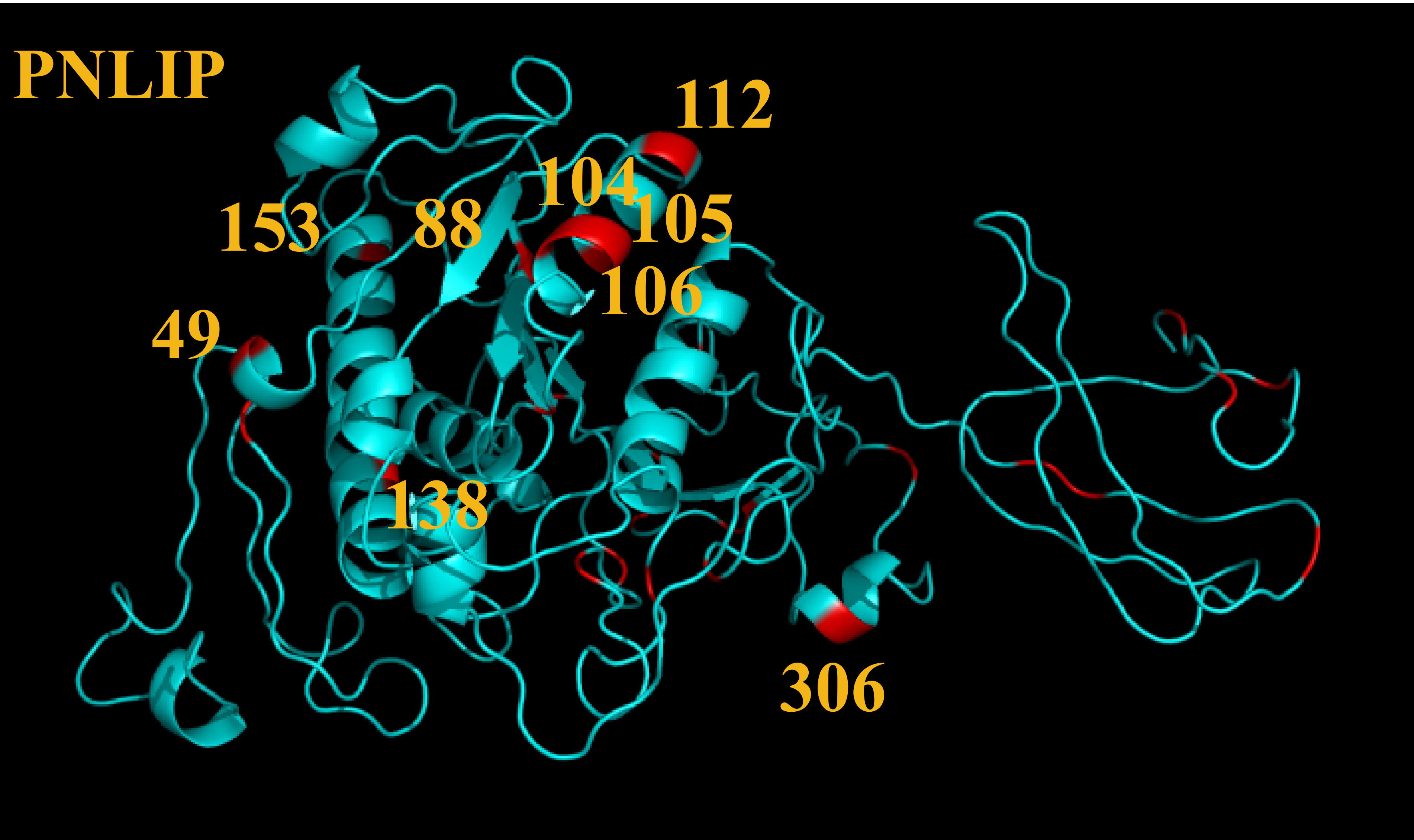
PNLIP
112
104
153
88
105
106
49
138
306

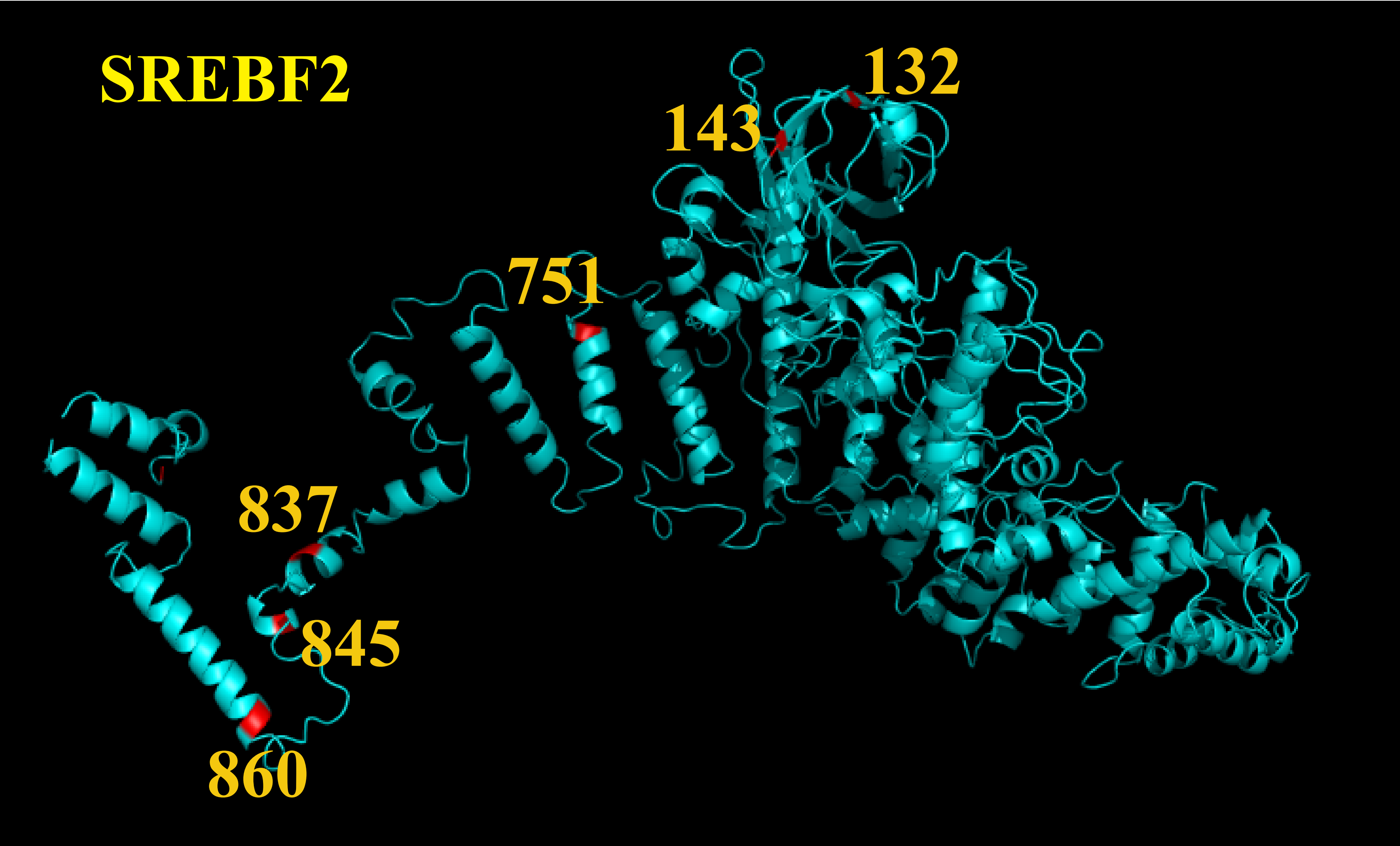
SREBF2
132
143
751
837
845
860
